# Supplementary material for: Bacterial RNA sensing by TLR8 requires RNase 6 processing and is inhibited by RNA 2’O-methylation
Source: EMBO Rep. 2024 Oct 3;25(11):4674–92. doi: 10.1038/s44319-024-00281-9 (PMC11549399; doi:10.1038/s44319-024-00281-9)
Supplement: Supplementary file 1 — Appendix [file 44319_2024_281_MOESM1_ESM.pdf]

Appendix for manuscript:

"Bacterial RNA sensing by TLR8 requires RNase 6 processing and is inhibited by RNA 2'O-methylation"

Table of Contents

Appendix Table S1. sgRNA sequences used for editing by CRISPR-Cas9 3

Appendix Table S2. qPCR primers 4

Appendix Table S1. sgRNA sequences used for editing by CRISPR-Cas9

Cell Type	Label	Sequence	Strand	Source
BLaER1	RNASE6_sgRNA_78	TGAGCCTTGGTGAGACGCTT	Negative	Microsynth
	RNASE6_sgRNA_85	TTGGCCTAAGCGTCTCACCA	Positive	Microsynth
	RNASE6_sgRNA_132	ATTGCCCTGTTGCATTGGAG	Negative	Microsynth
Primary human $CD14^+$ monocytes	RNASE6_sgRNA1_754	CAAGCAUGAAGUGGACACAC	Positive	Synthego
	RNASE6_sgRNA2_875	AUUUUGAUGCUUACAGUGCU	Positive	Synthego
	RNASE6_sgRNA3_039	GCAGCACUAUAGCGGCACUG	Positive	Synthego

Appendix Table S2. qPCR primers

Gene	Forward sequence	Reverse sequence	Source
RNASE6	CCCAACACTGAGACCAGAAAA	GTTGATGCCACTCATTGCCC	Microsynth
RNASET2	CCATCAATTACTACCAAGTTG	ATTTGCCAGCCAGACTTCCT	Microsynth
RNASE2	AGCGCGGAGACTGGGAAAC	ATTGCATTGGTGCATTGCTGG	Microsynth
TLR8	TCCTTCAGTCGTCAATGCTG	CGTTTGGGGAACTTCCTGTA	Microsynth
IL6	CCGGGAACGAAAGAGAAGCT	GCGCTTGTGGAGAAGGAGTT	Microsynth
CXCL10	ATTTGCTGCCTTATCTTTCTG	TCTCACCCTTCTTTTTCATTGTAG	Microsynth
B2M	GTGCTCGCGCTACTCTCTCT	GTAAACTTCAATGTCGGATGG	Microsynth
