# Supplementary figures and images for: Bacterial RNA sensing by TLR8 requires RNase 6 processing and is inhibited by RNA 2’O-methylation
Source: EMBO Rep. 2024 Oct 3;25(11):4674–92. doi: 10.1038/s44319-024-00281-9 (PMC11549399; doi:10.1038/s44319-024-00281-9)

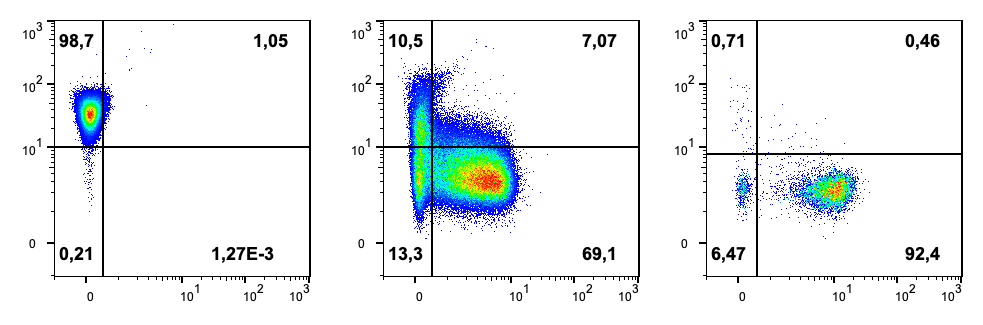

Supplement: Supplementary file 3 — Source data Fig. 1 [file 44319_2024_281_MOESM3_ESM.zip › Figure 1/1A/20240201-BLaER transdiff time-Layout-2.png]

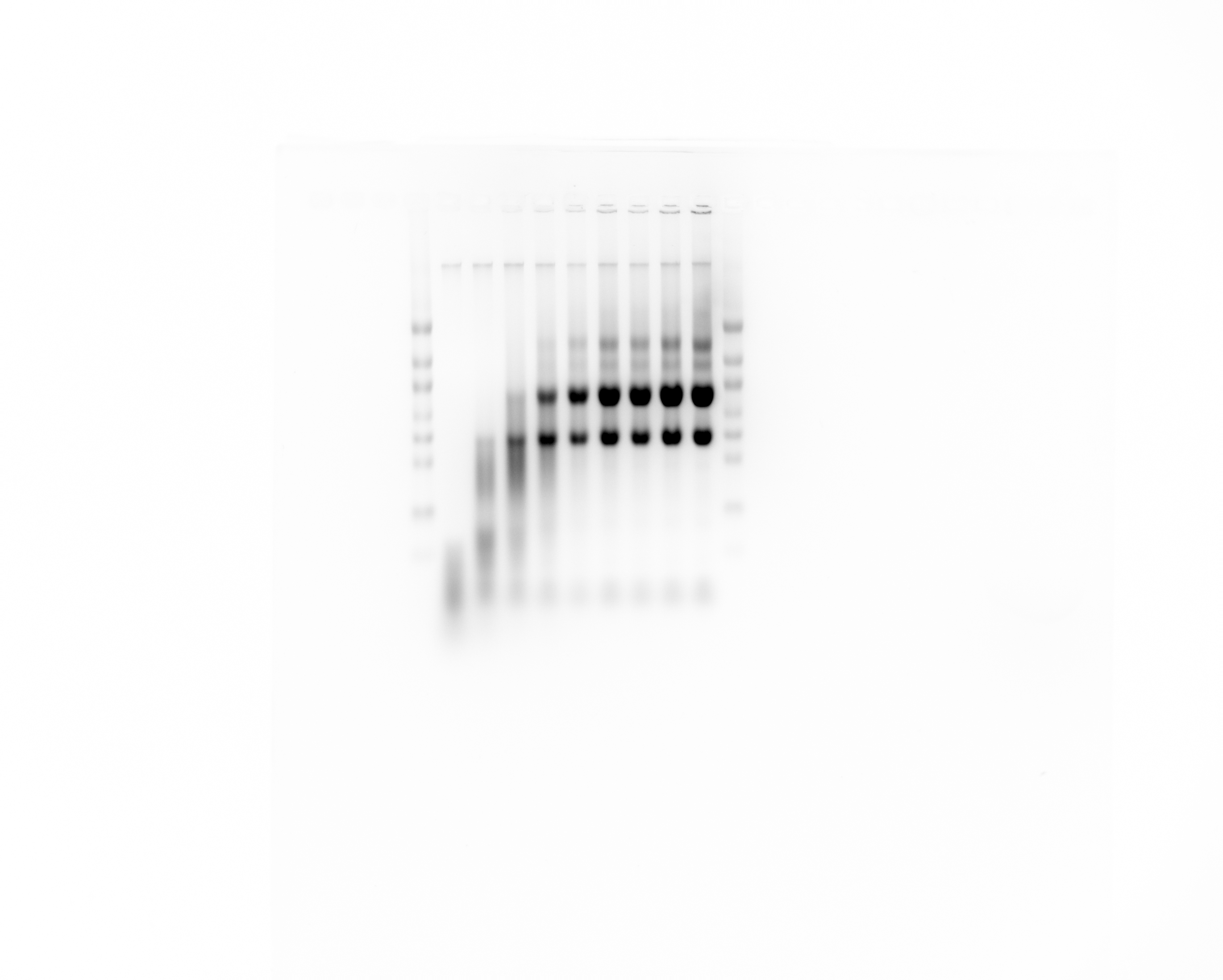

Supplement: Supplementary file 4 — Source data Fig. 2 [file 44319_2024_281_MOESM4_ESM.zip › Figure 2/2H/2H.tif]

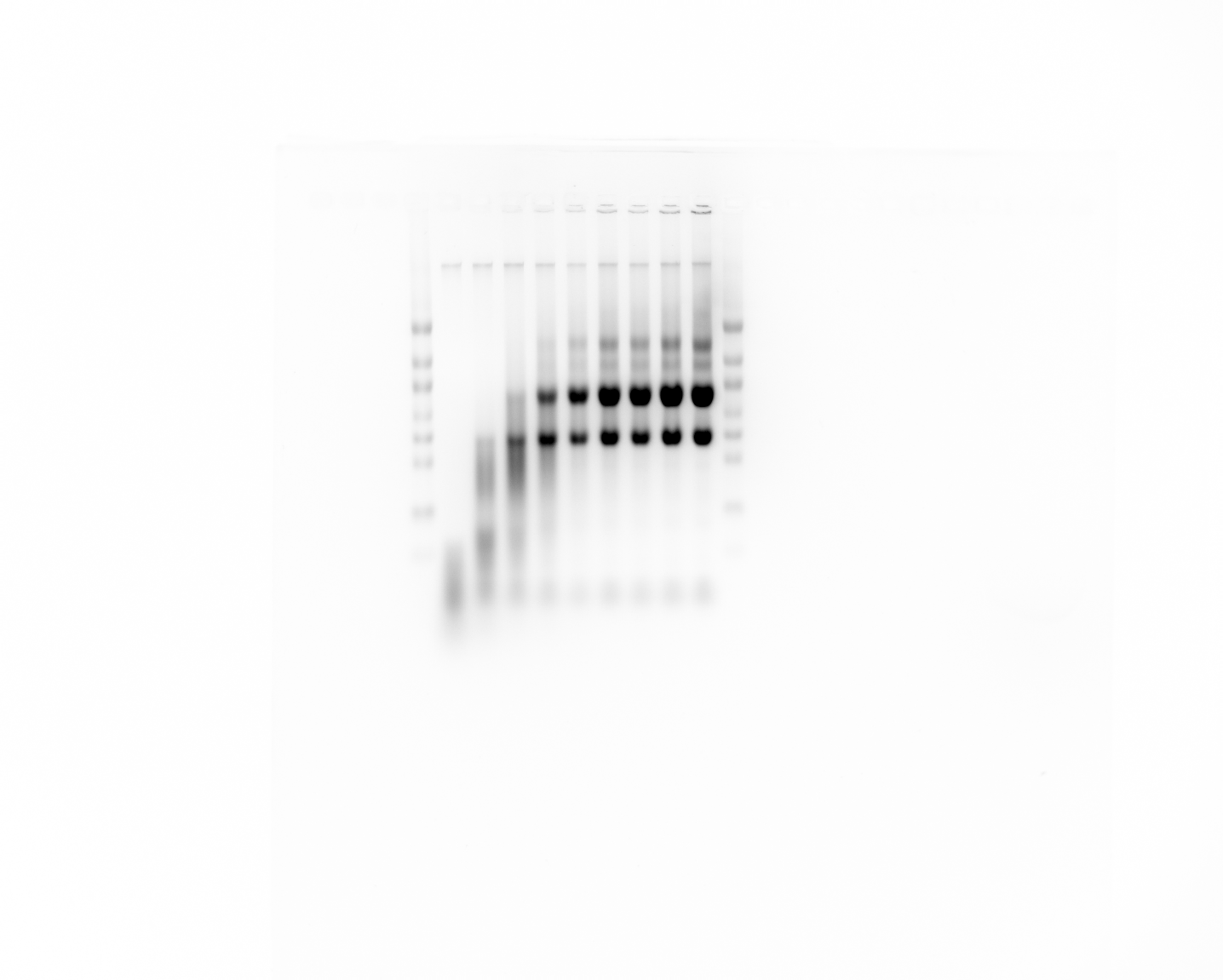


Figure 2H

500

1000

1500

2000

3000

4000

6000

Bases

RNase 6 (ng)

0.1

0.25

0.5

1

2

4

8

16

Ladder

Loading control

Ladder

Supplement: Supplementary file 4 — Source data Fig. 2 [file 44319_2024_281_MOESM4_ESM.zip › Figure 2/2H/2H.docx]

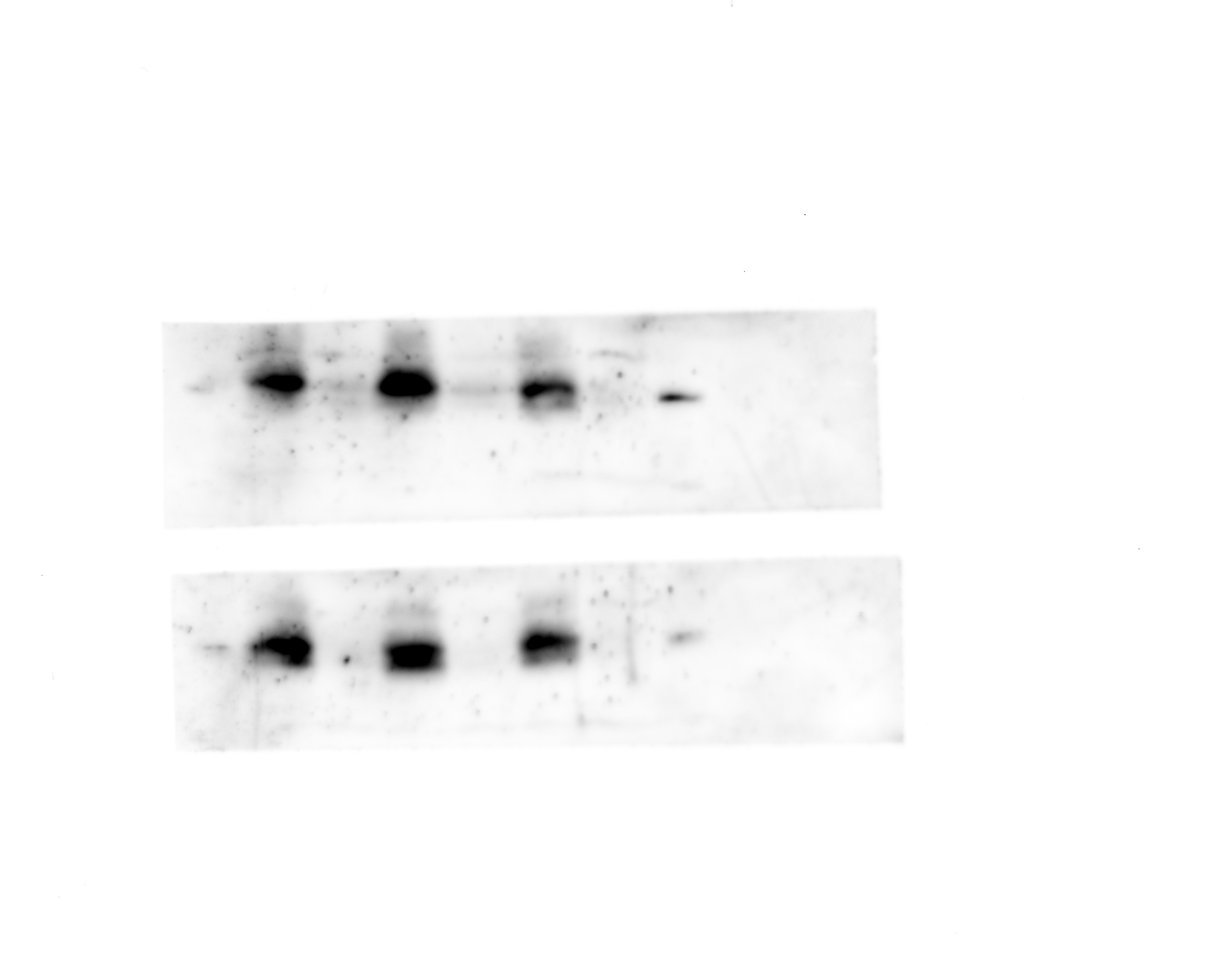

Supplement: Supplementary file 4 — Source data Fig. 2 [file 44319_2024_281_MOESM4_ESM.zip › Figure 2/2F/Western blot_RNase 6.tif]

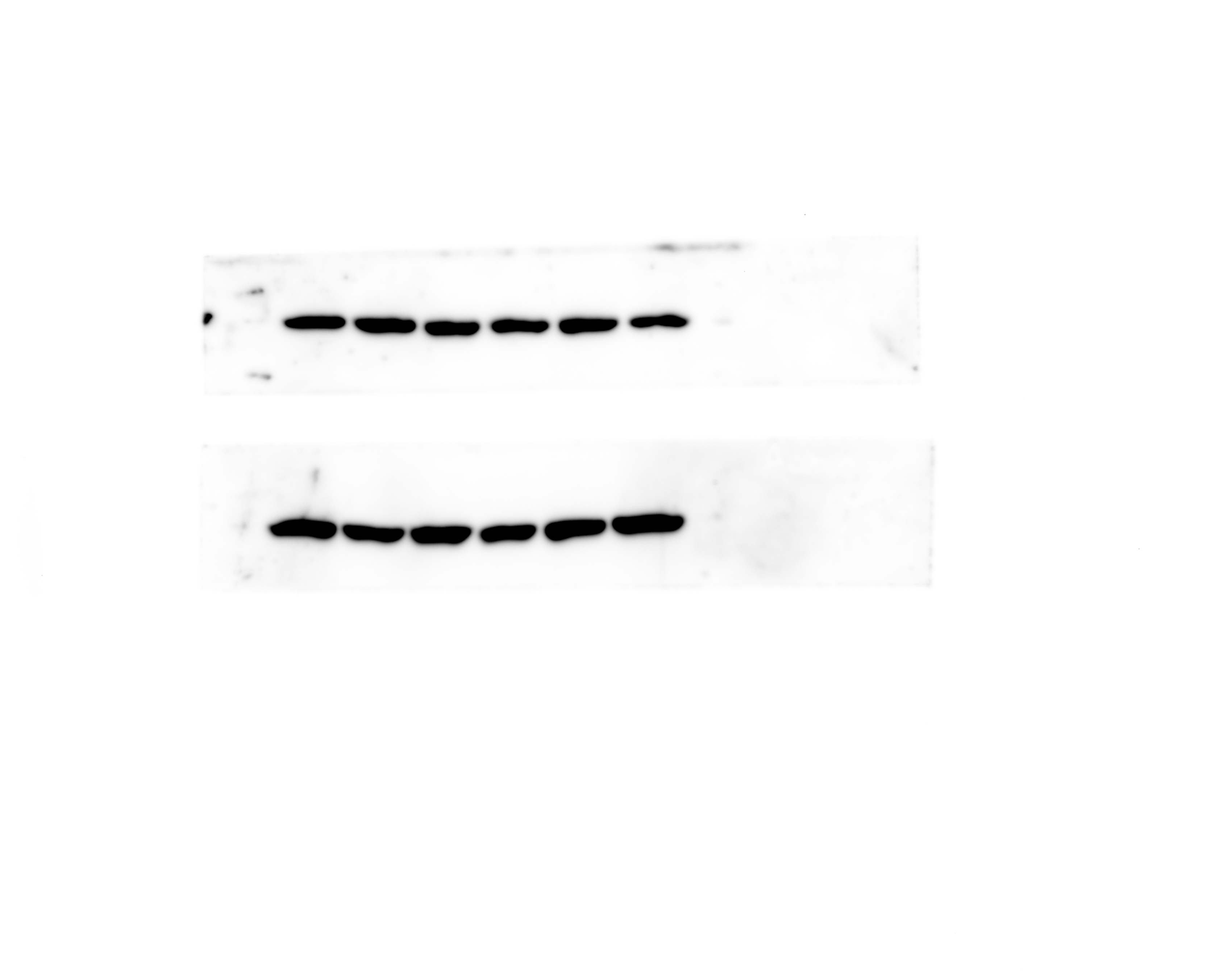

Supplement: Supplementary file 4 — Source data Fig. 2 [file 44319_2024_281_MOESM4_ESM.zip › Figure 2/2F/Western blot_Beta-actin.tif]

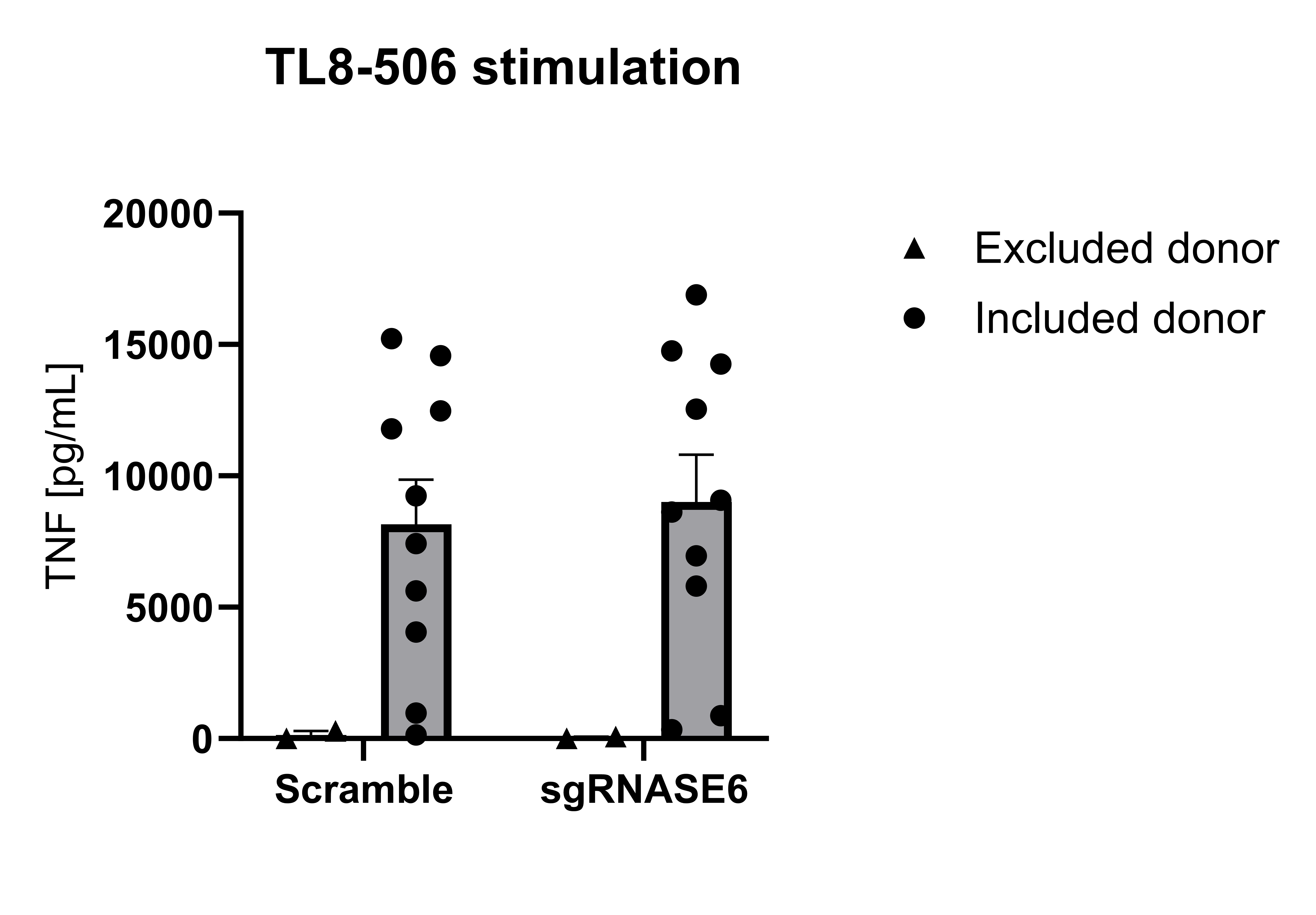

Supplement: Supplementary file 4 — Source data Fig. 2 [file 44319_2024_281_MOESM4_ESM.zip › Figure 2/2F/Excluded donor.tif]

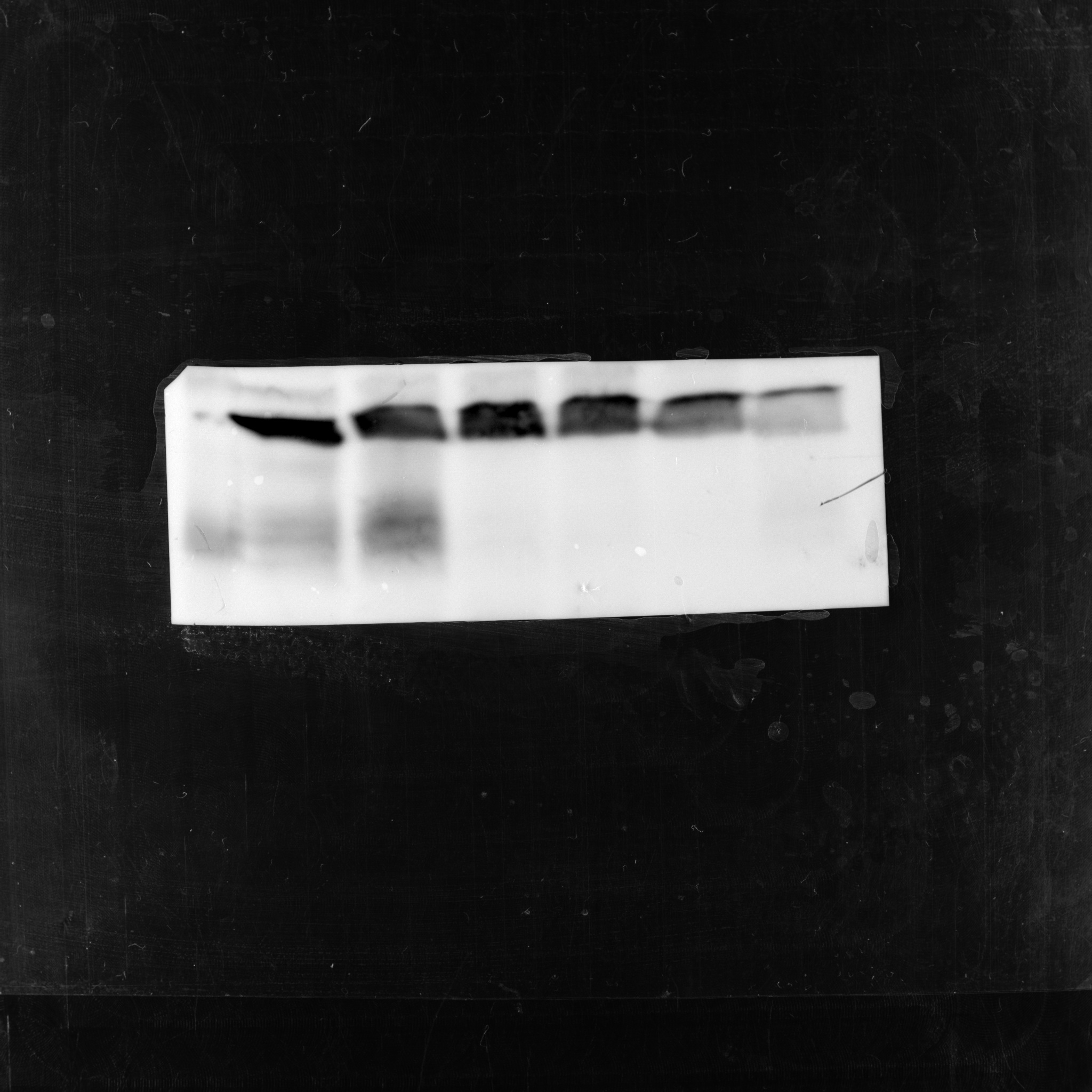

Supplement: Supplementary file 4 — Source data Fig. 2 [file 44319_2024_281_MOESM4_ESM.zip › Figure 2/2B/Western blot_RNase 6.tif]

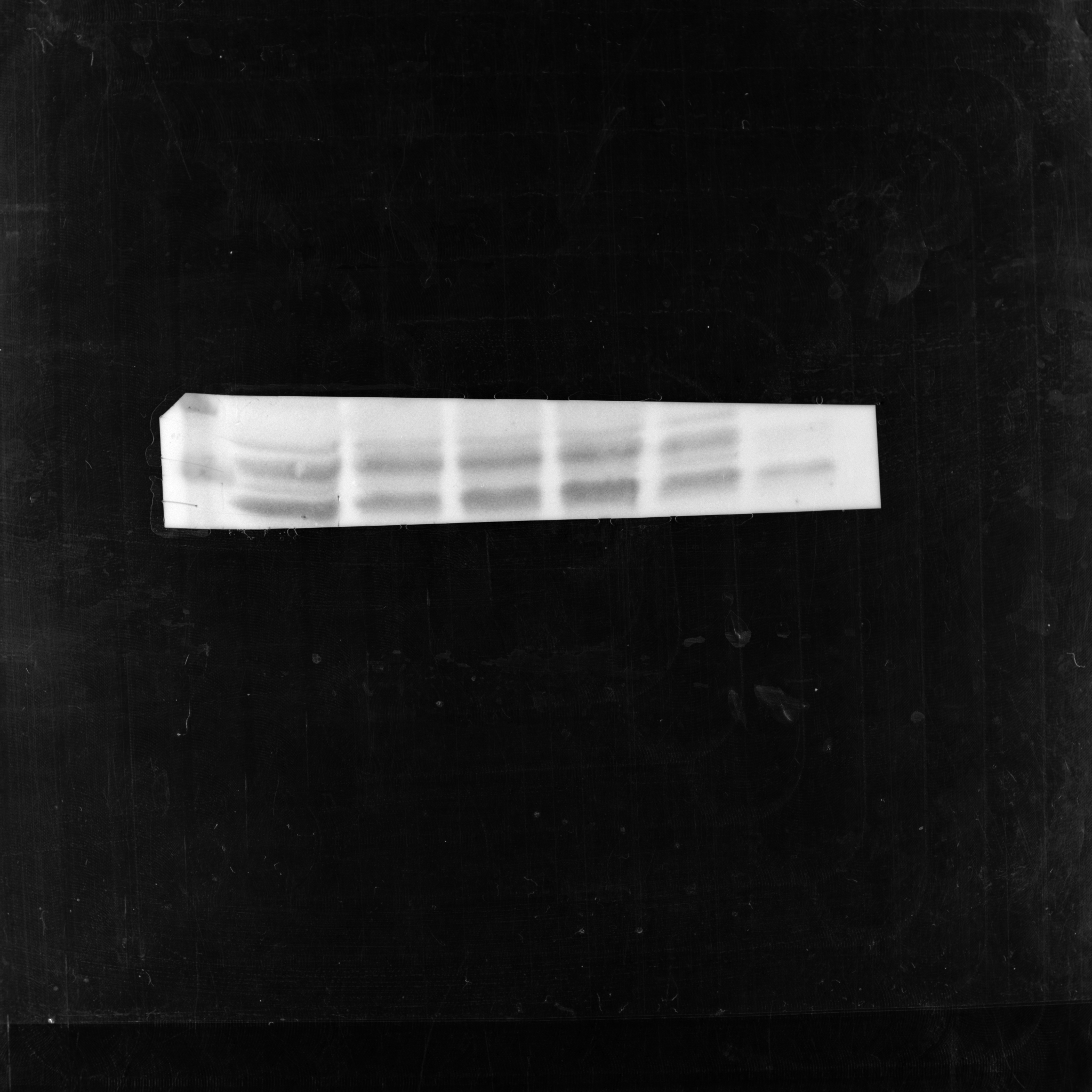

Supplement: Supplementary file 4 — Source data Fig. 2 [file 44319_2024_281_MOESM4_ESM.zip › Figure 2/2B/Western blot_RNase T2.tif]

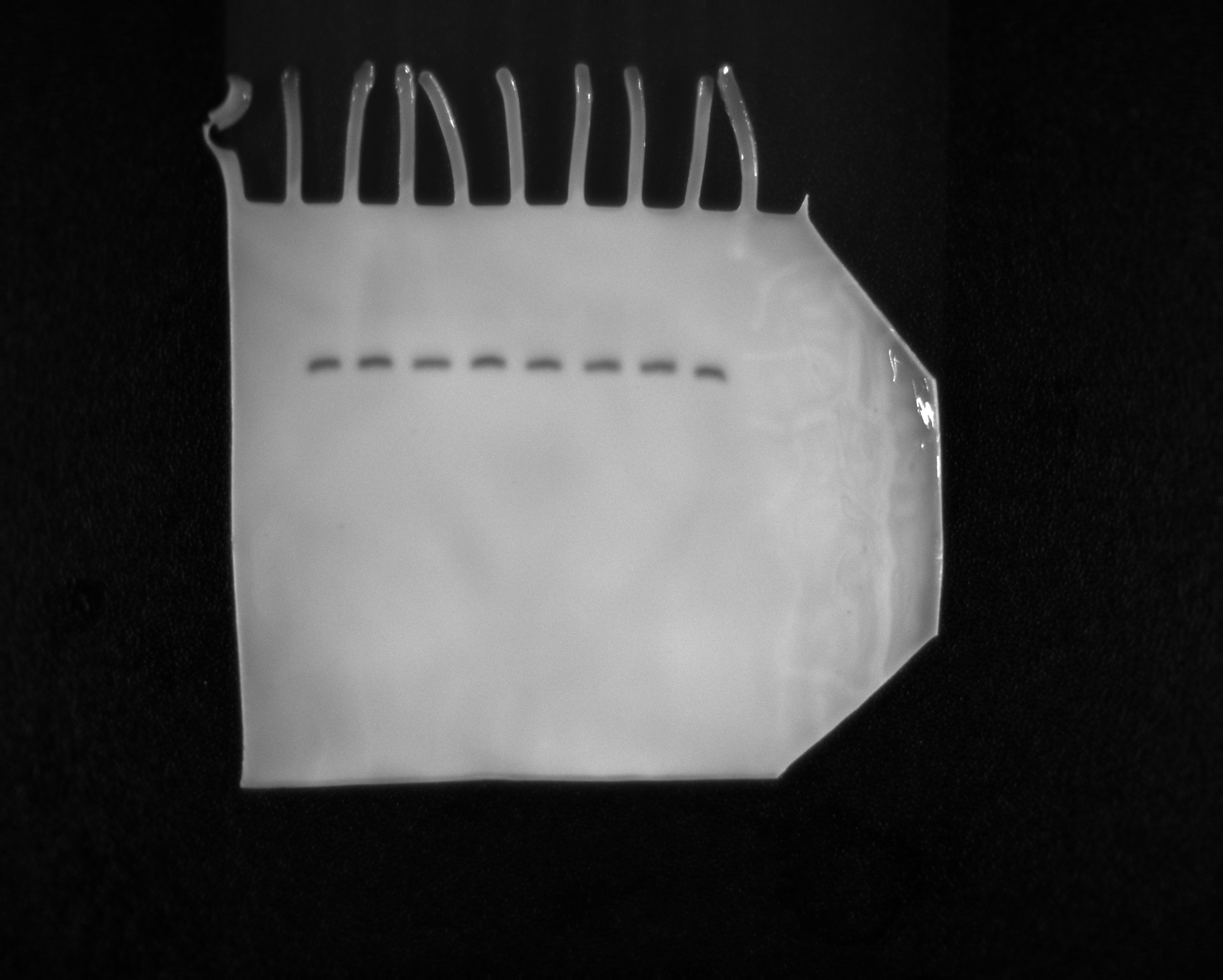

Supplement: Supplementary file 5 — Source data Fig. 3 [file 44319_2024_281_MOESM5_ESM.zip › Figure 3/3E/3E_rUdA RT2.tif]

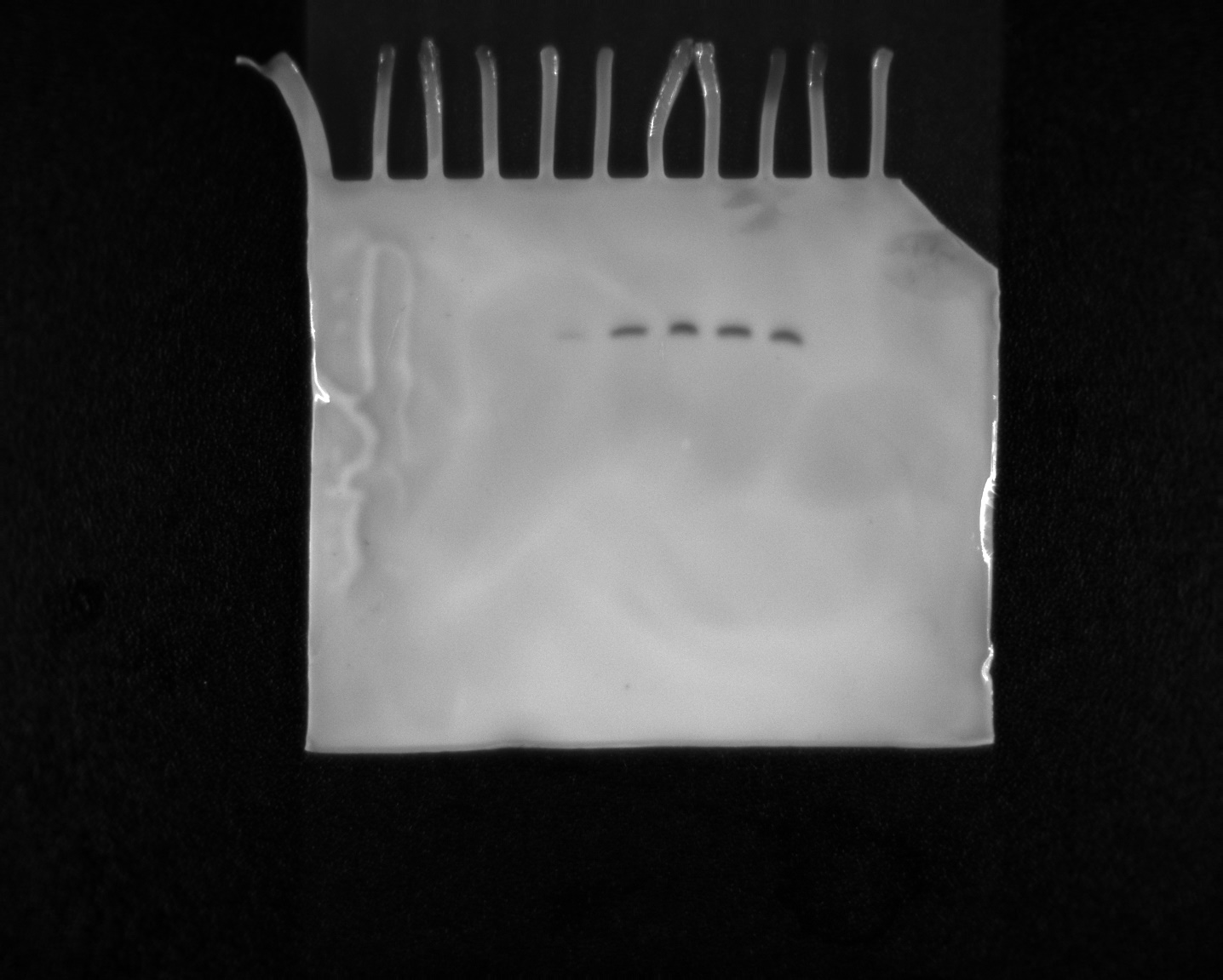


RNase 6 (ng/µl)

0.05

0.1

0.25

0.5

1

2.5

Loading control

(dAdC)3U(dAdC)4

Figure 3E

5

Supplement: Supplementary file 5 — Source data Fig. 3 [file 44319_2024_281_MOESM5_ESM.zip › Figure 3/3E/3E_rUdA R6.docx]

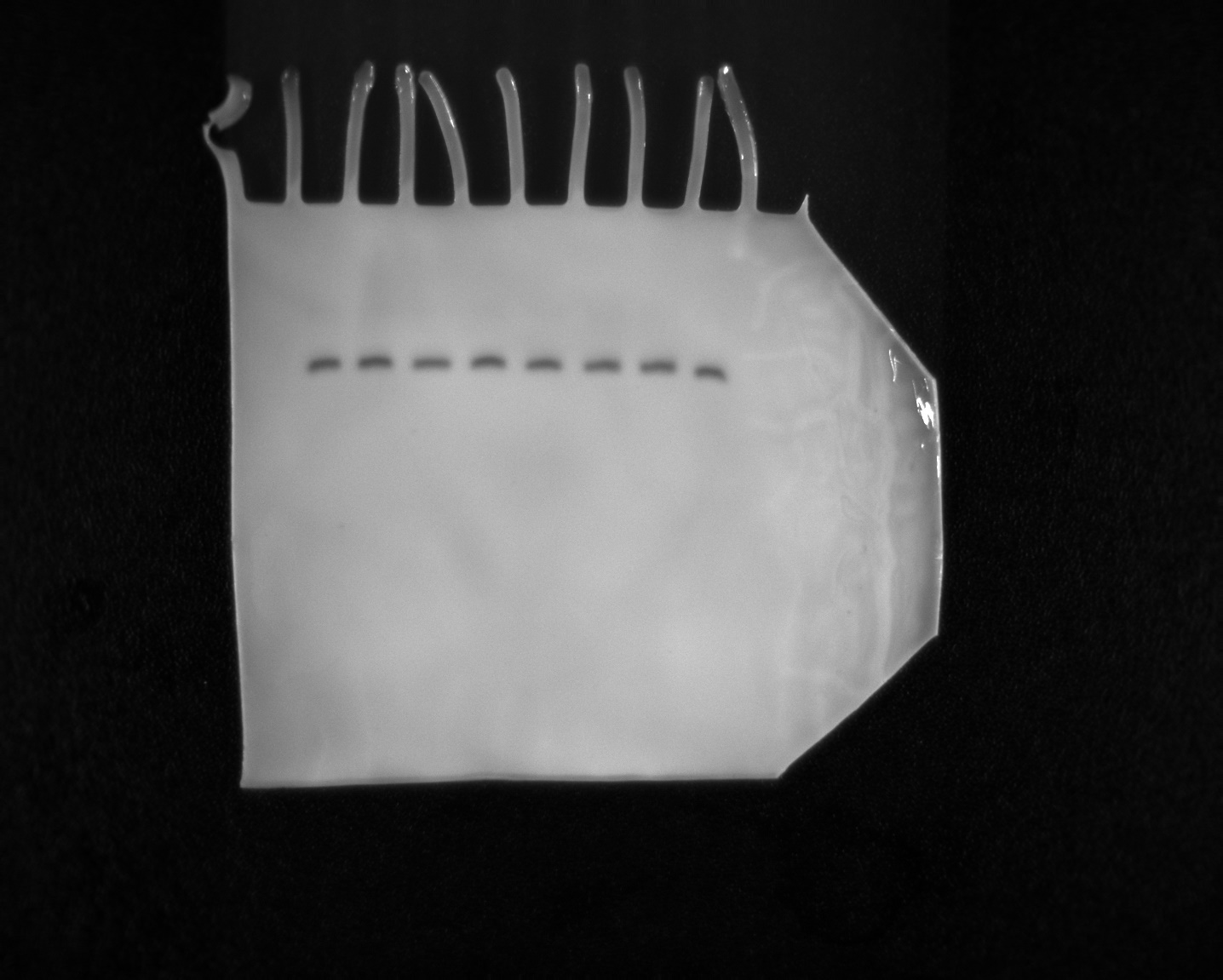


RNase T2 (ng/µl)

Loading control

0.05

0.1

0.25

0.5

1

2.5

5

(dAdC)3U(dAdC)4

Figure 3E

Supplement: Supplementary file 5 — Source data Fig. 3 [file 44319_2024_281_MOESM5_ESM.zip › Figure 3/3E/3E_rUdA RT2.docx]

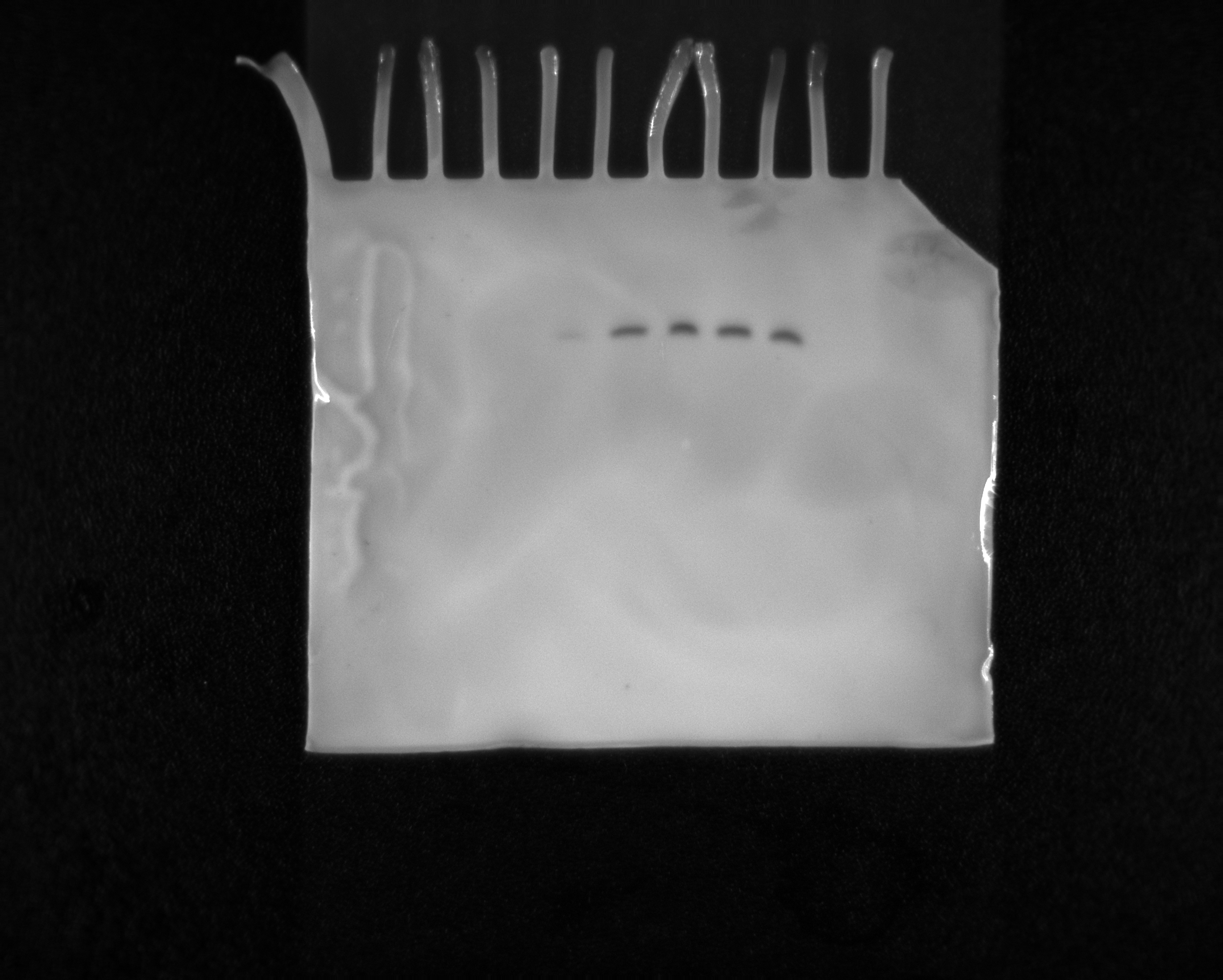

Supplement: Supplementary file 5 — Source data Fig. 3 [file 44319_2024_281_MOESM5_ESM.zip › Figure 3/3E/3E_rUdA R6.tif]

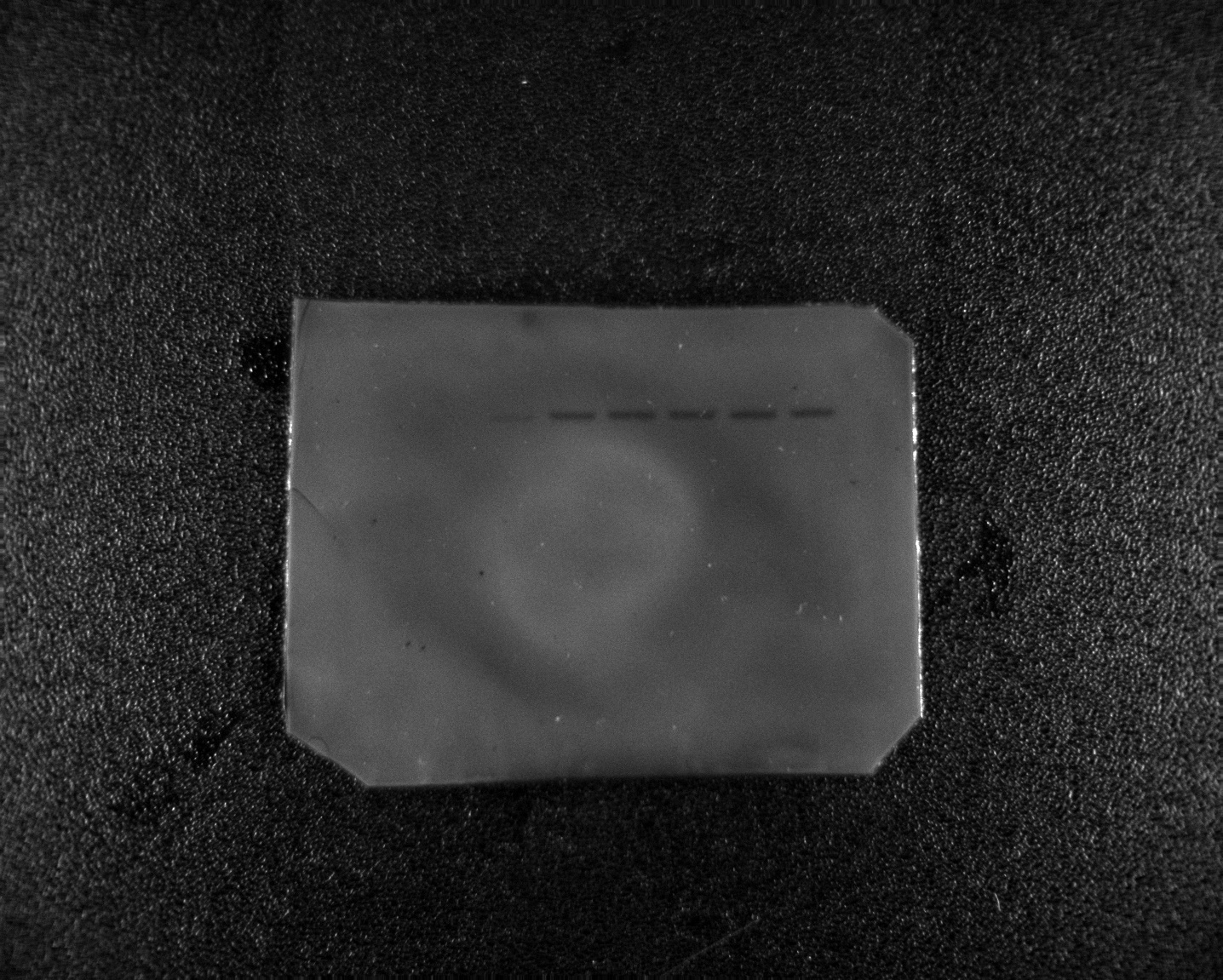


(dAdC)3UUUU(dAdC)4

RNase 6 (ng/µl)

5

2.5

1

0.5

0.25

0.1

0.05

Loading control

Figure 3C

Supplement: Supplementary file 5 — Source data Fig. 3 [file 44319_2024_281_MOESM5_ESM.zip › Figure 3/3C/3C_UUUU.docx]

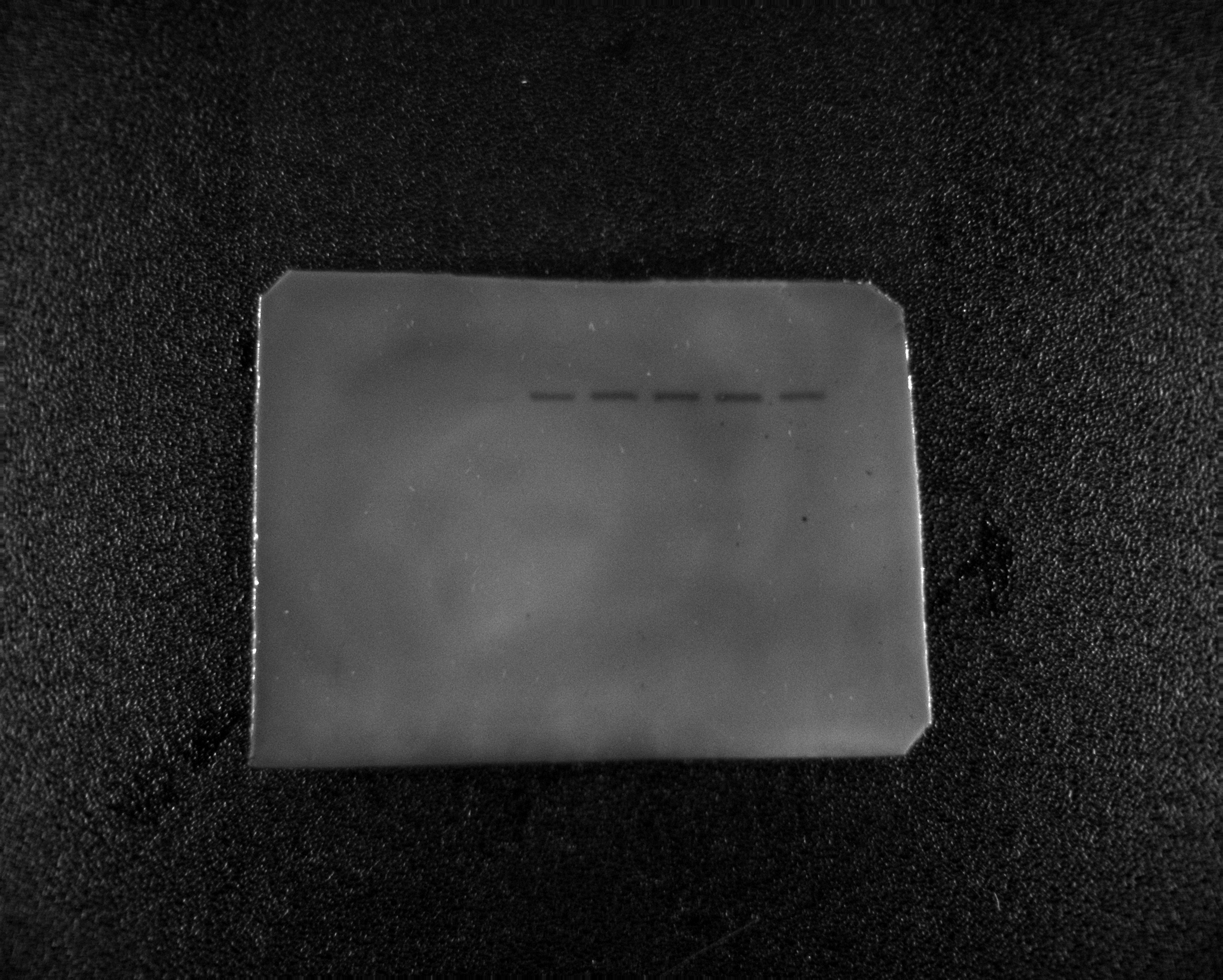

Supplement: Supplementary file 5 — Source data Fig. 3 [file 44319_2024_281_MOESM5_ESM.zip › Figure 3/3C/3C_UUCC.tif]

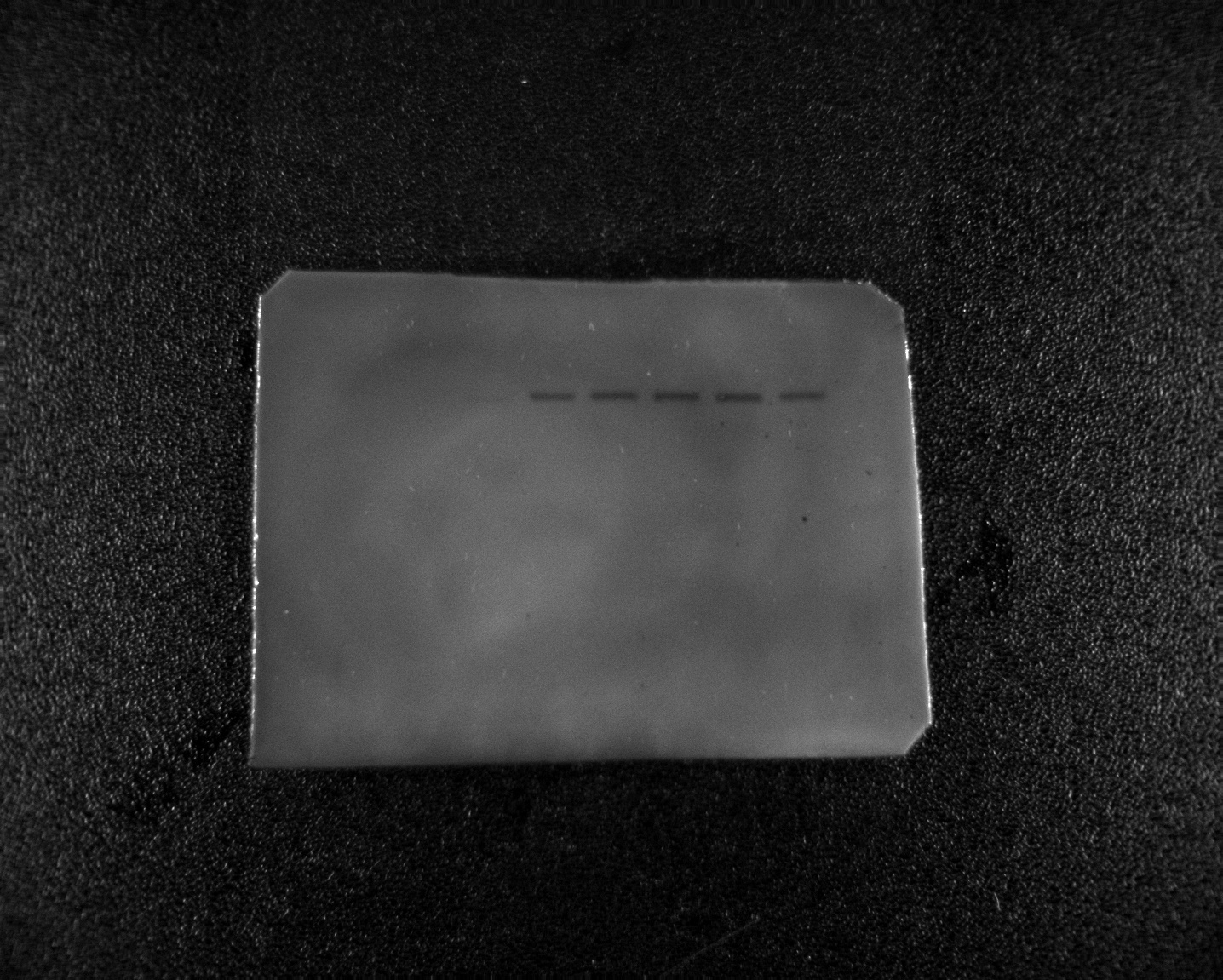


(dAdC)3UUCC(dAdC)4

5

2.5

1

0.5

0.25

0.1

Loading control

0.05

RNase 6 (ng/µl)

Figure 3C

Supplement: Supplementary file 5 — Source data Fig. 3 [file 44319_2024_281_MOESM5_ESM.zip › Figure 3/3C/3C_UUCC.docx]

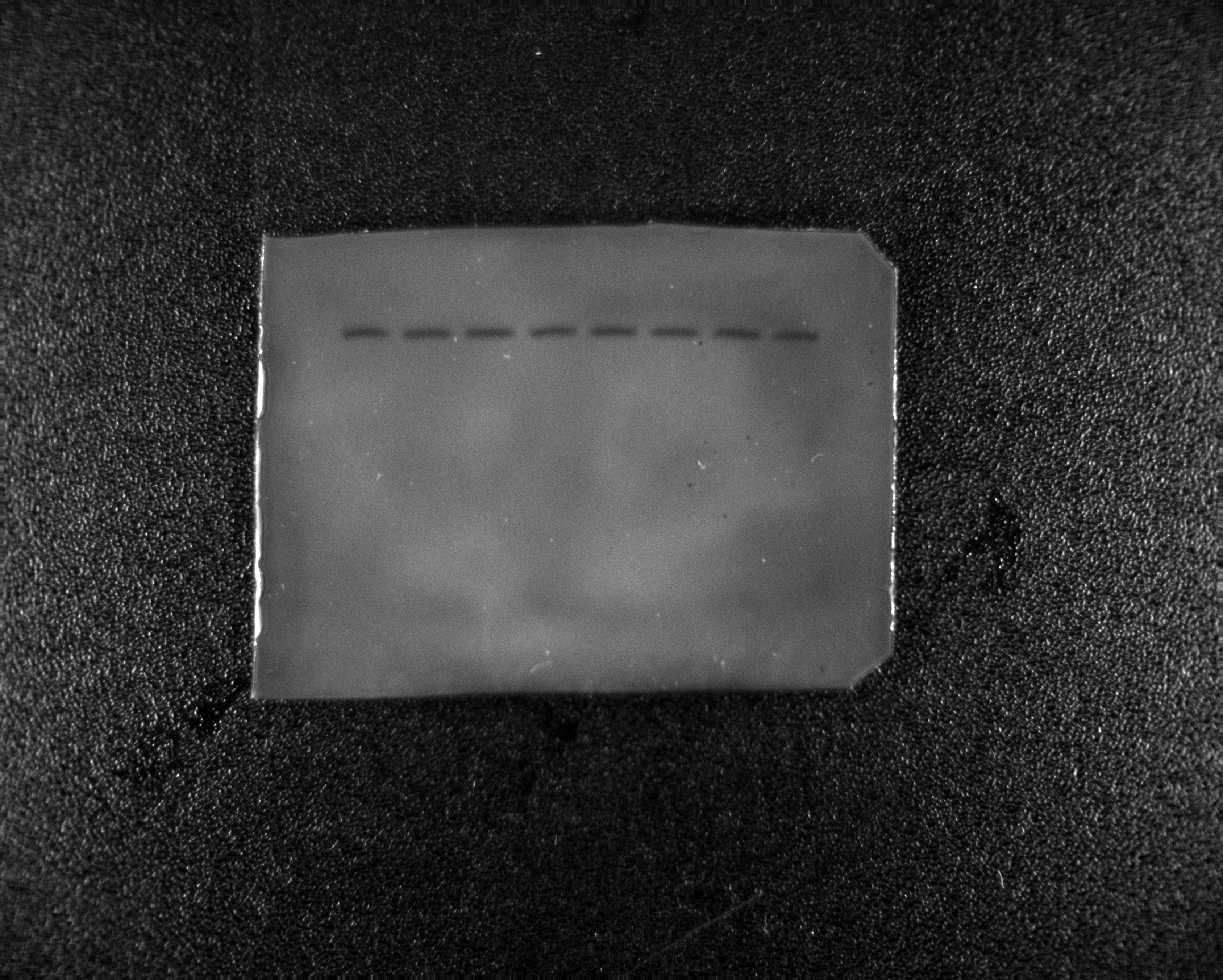

Supplement: Supplementary file 5 — Source data Fig. 3 [file 44319_2024_281_MOESM5_ESM.zip › Figure 3/3C/3C_UUGG.tif]

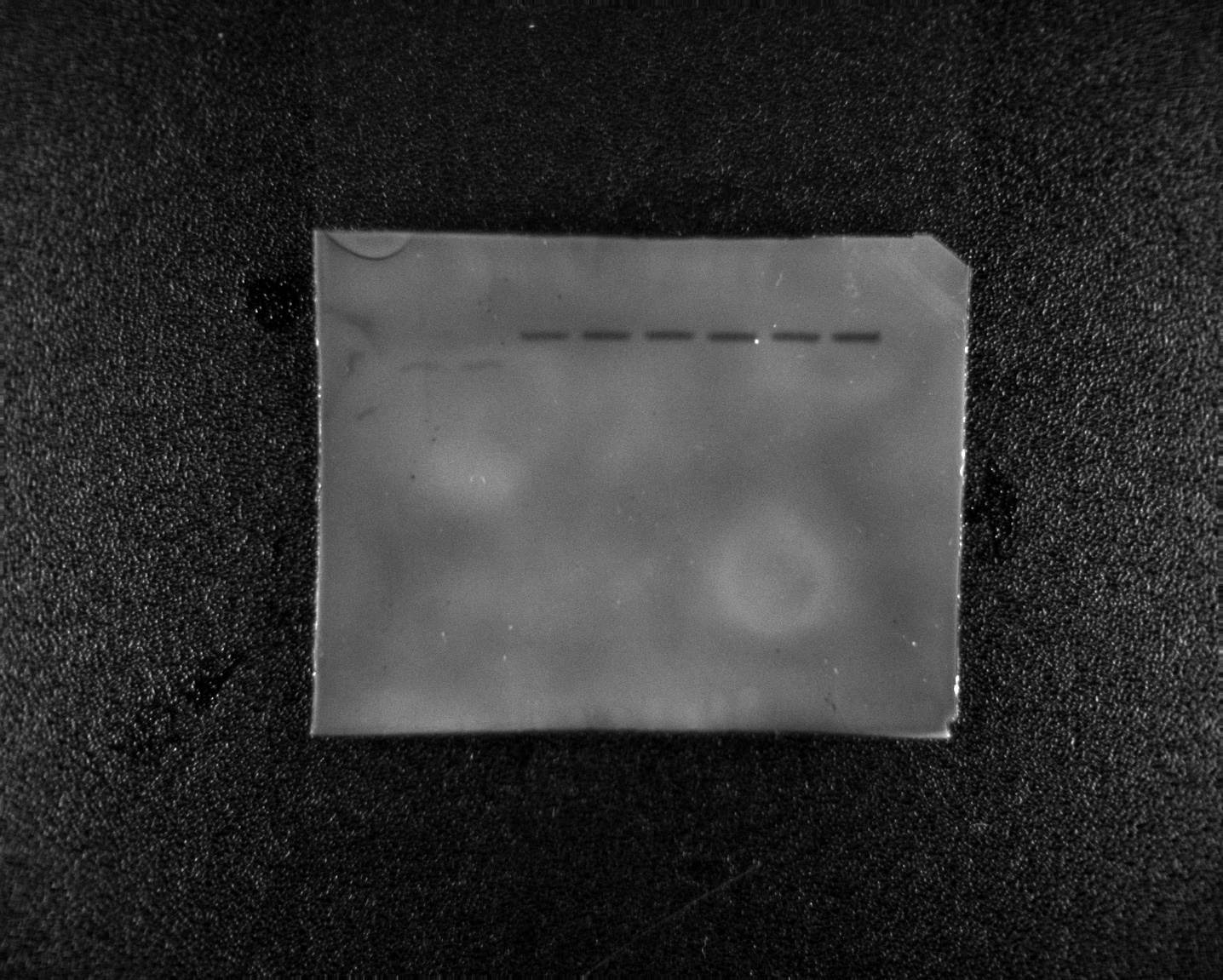


(dAdC)3UUAA(dAdC)4

Figure 3C

RNase 6 (ng/µl)

0.05

0.1

0.25

0.5

1

2.5

5

Loading control

Supplement: Supplementary file 5 — Source data Fig. 3 [file 44319_2024_281_MOESM5_ESM.zip › Figure 3/3C/3C_UUAA.docx]

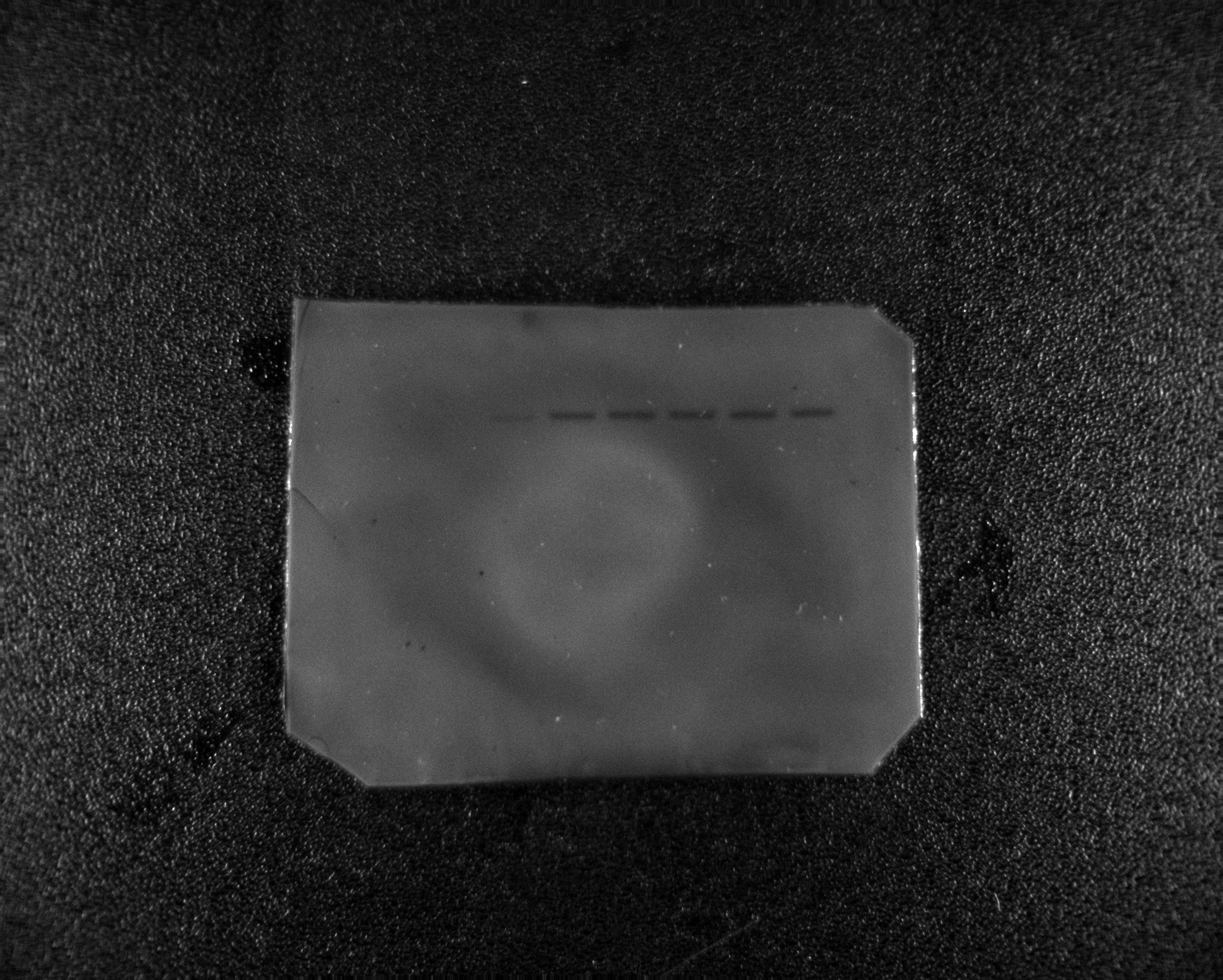

Supplement: Supplementary file 5 — Source data Fig. 3 [file 44319_2024_281_MOESM5_ESM.zip › Figure 3/3C/3C_UUUU.tif]

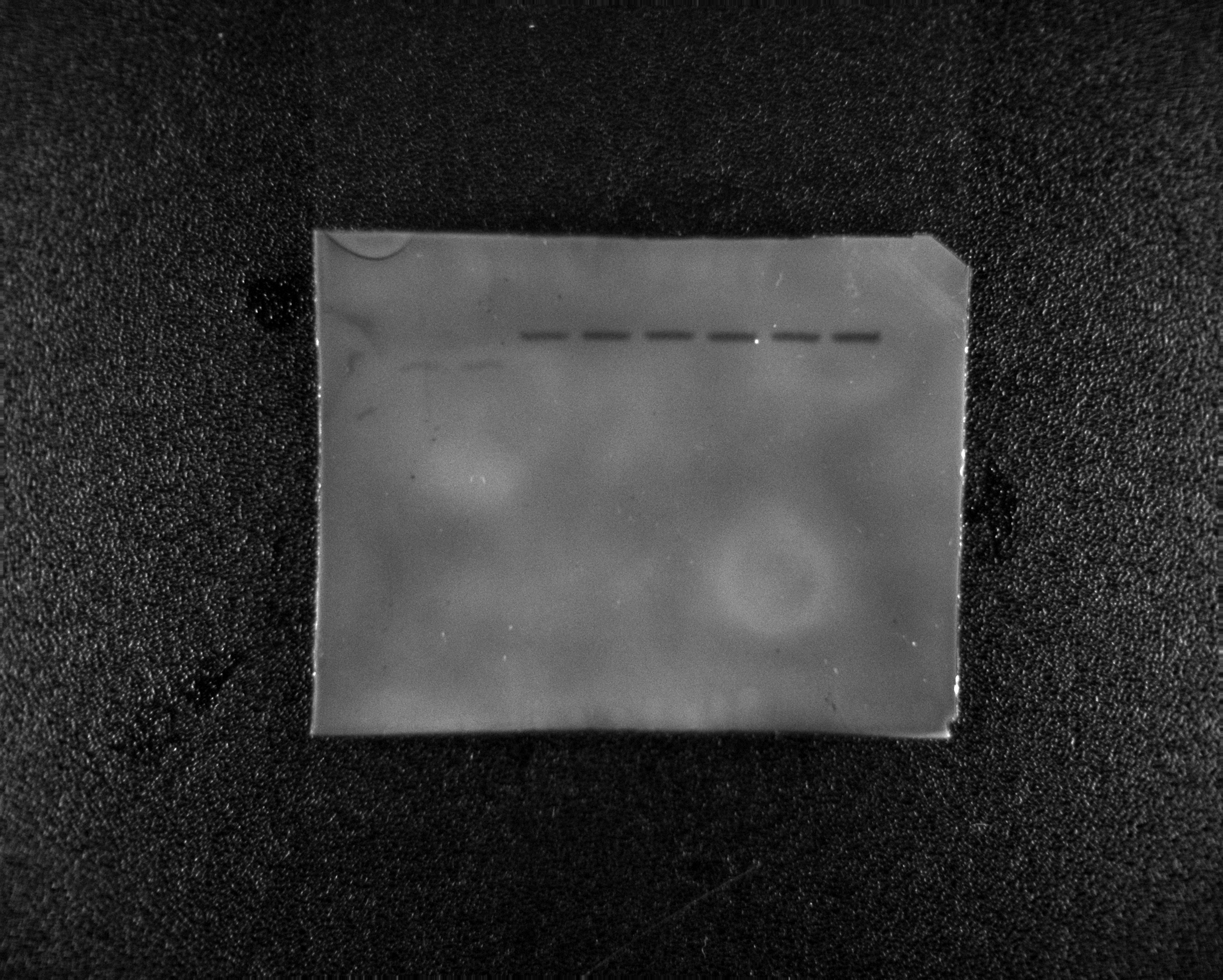

Supplement: Supplementary file 5 — Source data Fig. 3 [file 44319_2024_281_MOESM5_ESM.zip › Figure 3/3C/3C_UUAA.tif]

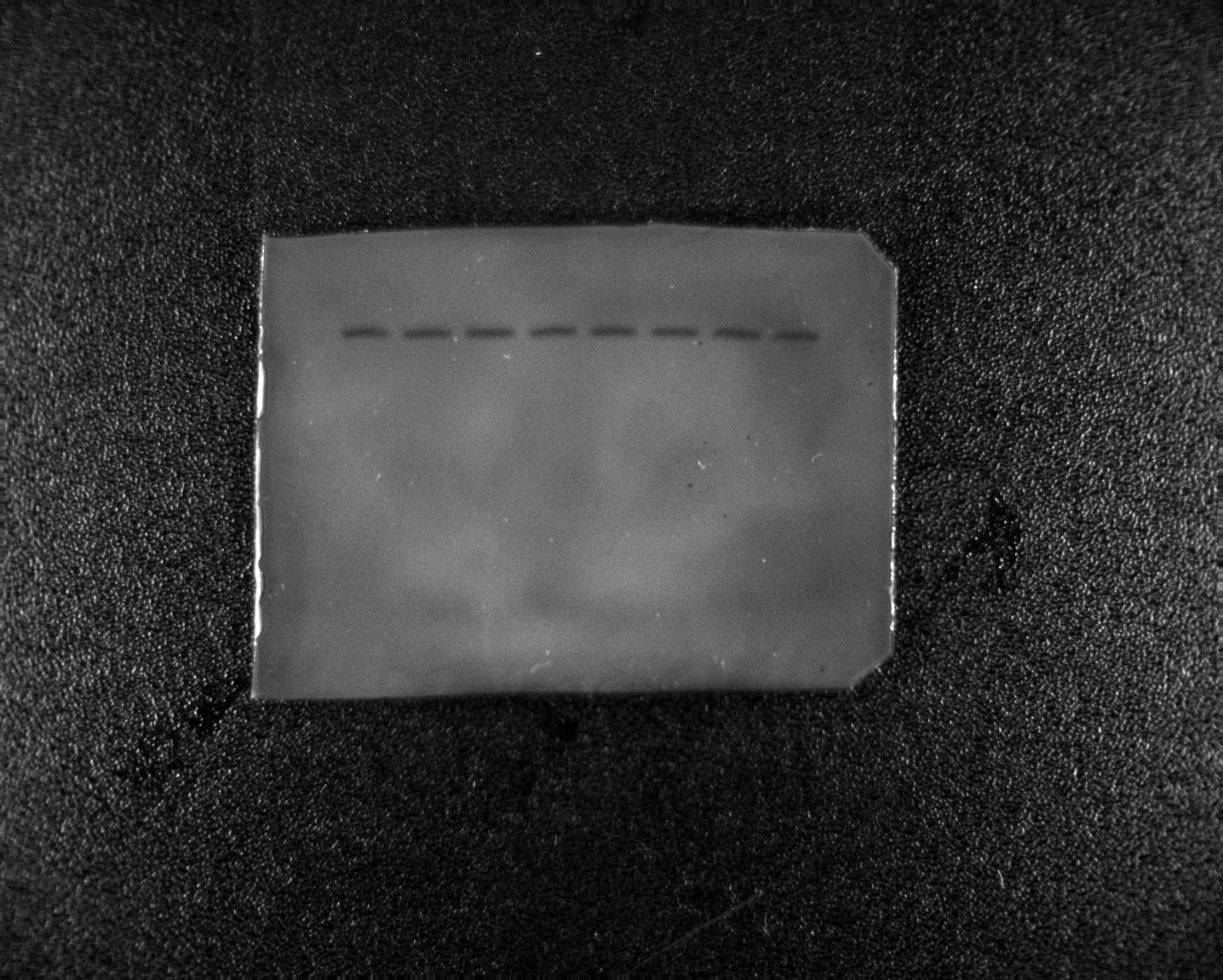


Loading control

5

2.5

1

0.5

0.25

0.1

0.05

RNase 6 (ng/µl)

(dAdC)3UUGG(dAdC)4

Figure 3C

Supplement: Supplementary file 5 — Source data Fig. 3 [file 44319_2024_281_MOESM5_ESM.zip › Figure 3/3C/3C_UUGG.docx]

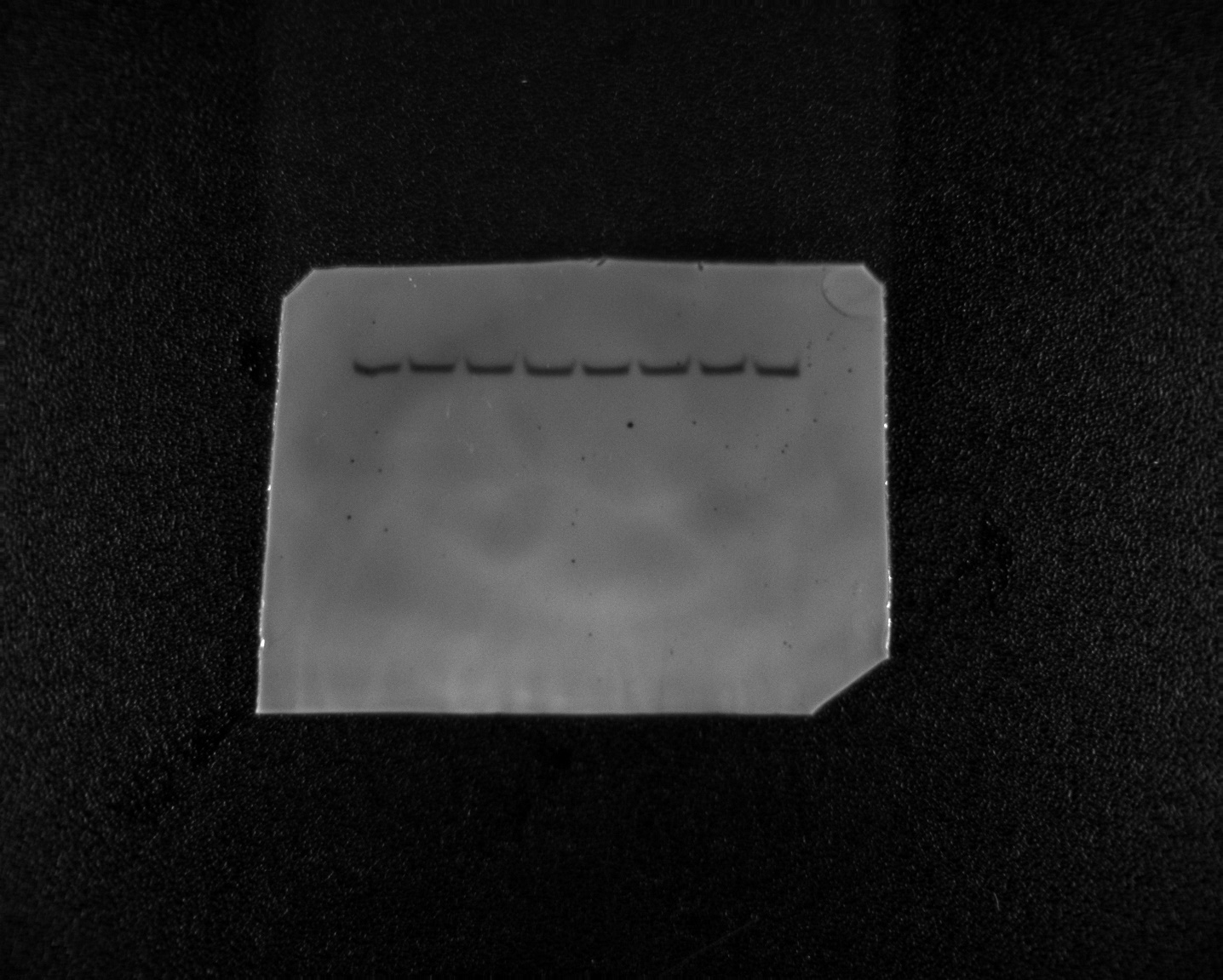


RNase T2 (ng/µl)

Loading control

0.05

0.1

0.25

1

0.5

2.5

5

(dAdC)3UUCC(dAdC)4

Figure 3D

Supplement: Supplementary file 5 — Source data Fig. 3 [file 44319_2024_281_MOESM5_ESM.zip › Figure 3/3D/3D_UUCC.docx]

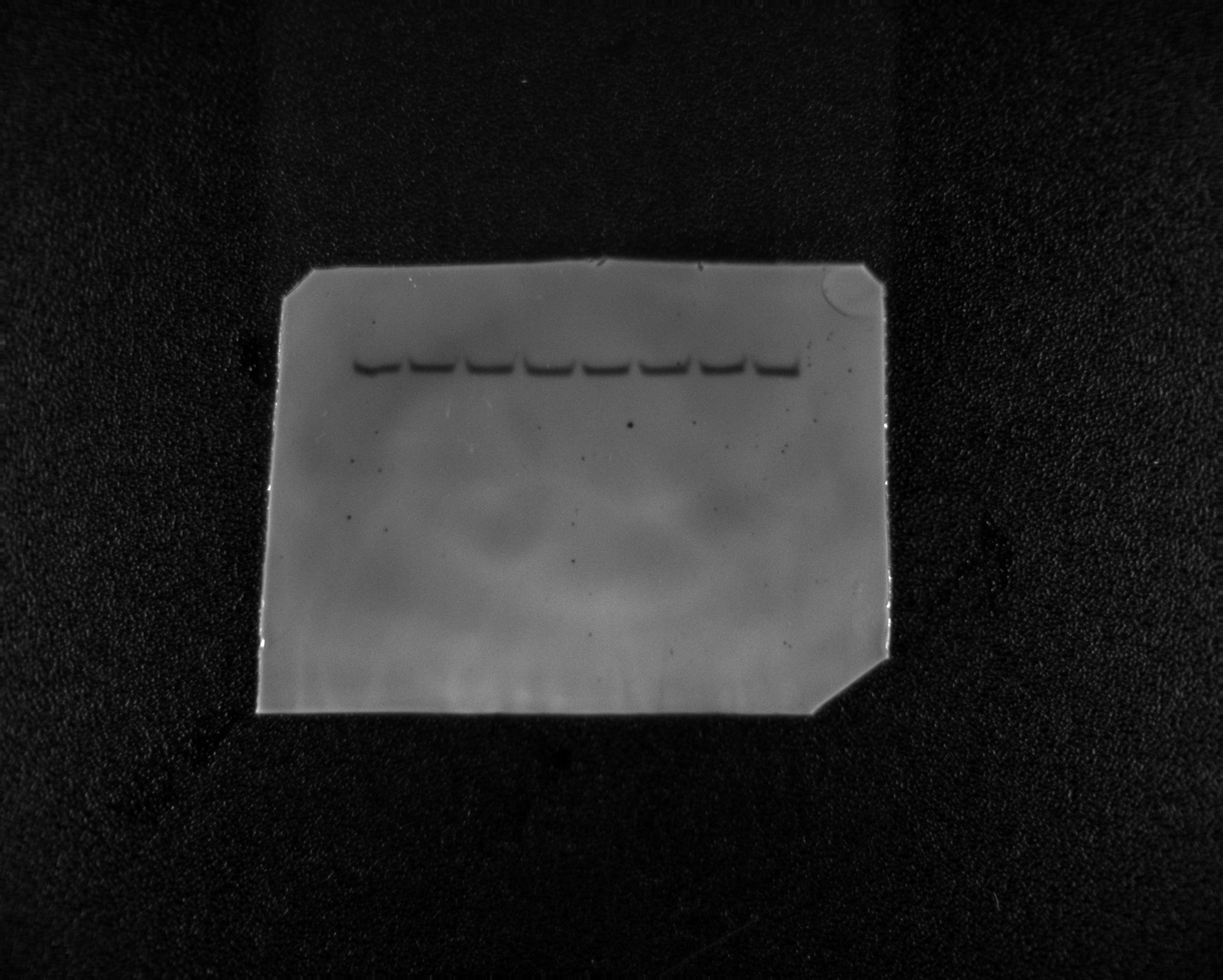

Supplement: Supplementary file 5 — Source data Fig. 3 [file 44319_2024_281_MOESM5_ESM.zip › Figure 3/3D/3D_UUCC.tif]

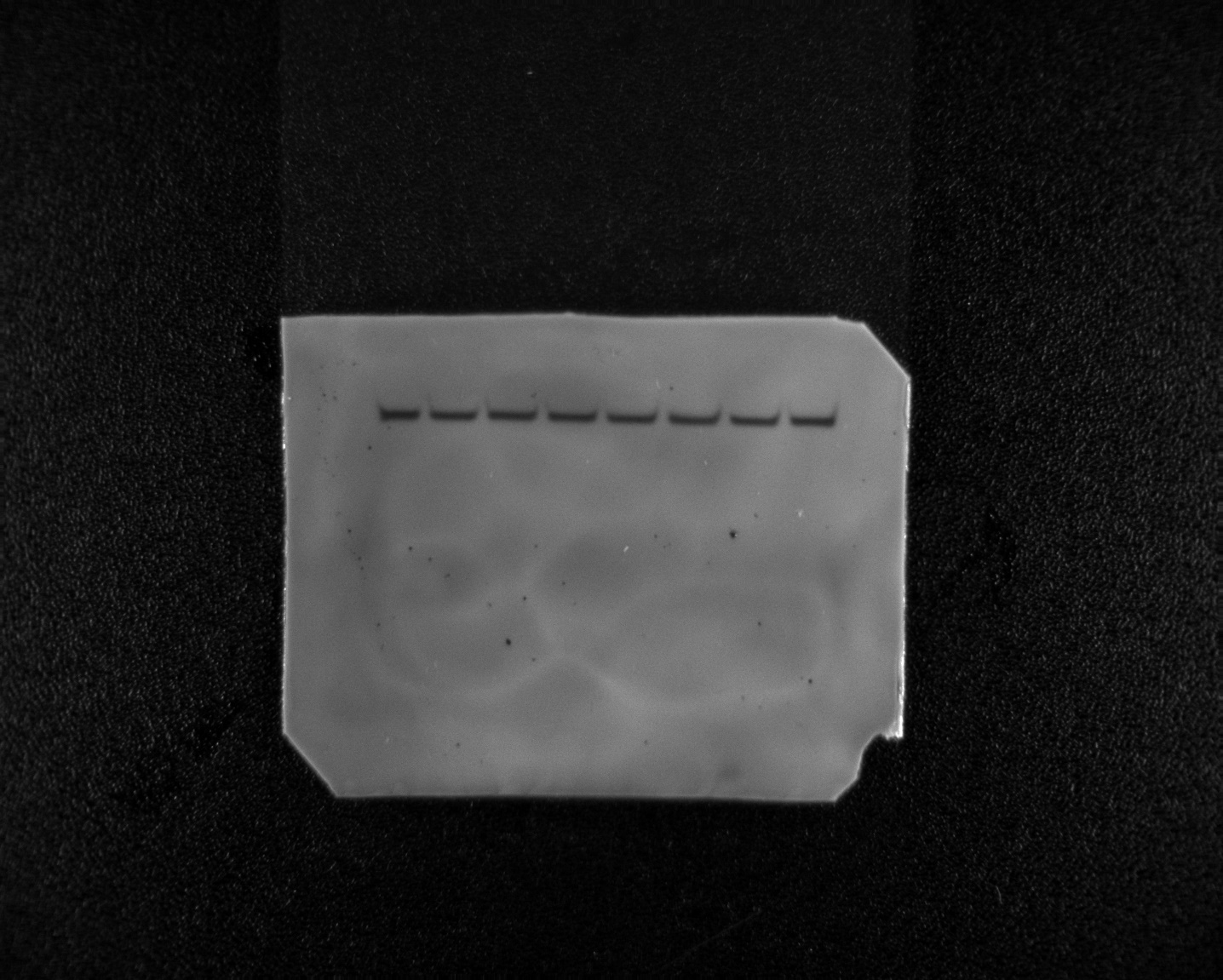


0.05

0.1

0.25

0.5

1

2.5

5

Loading control

RNase T2 (ng/µl)

Figure 3D

(dAdC)3UUUU(dAdC)4

Supplement: Supplementary file 5 — Source data Fig. 3 [file 44319_2024_281_MOESM5_ESM.zip › Figure 3/3D/3D_UUUU.docx]

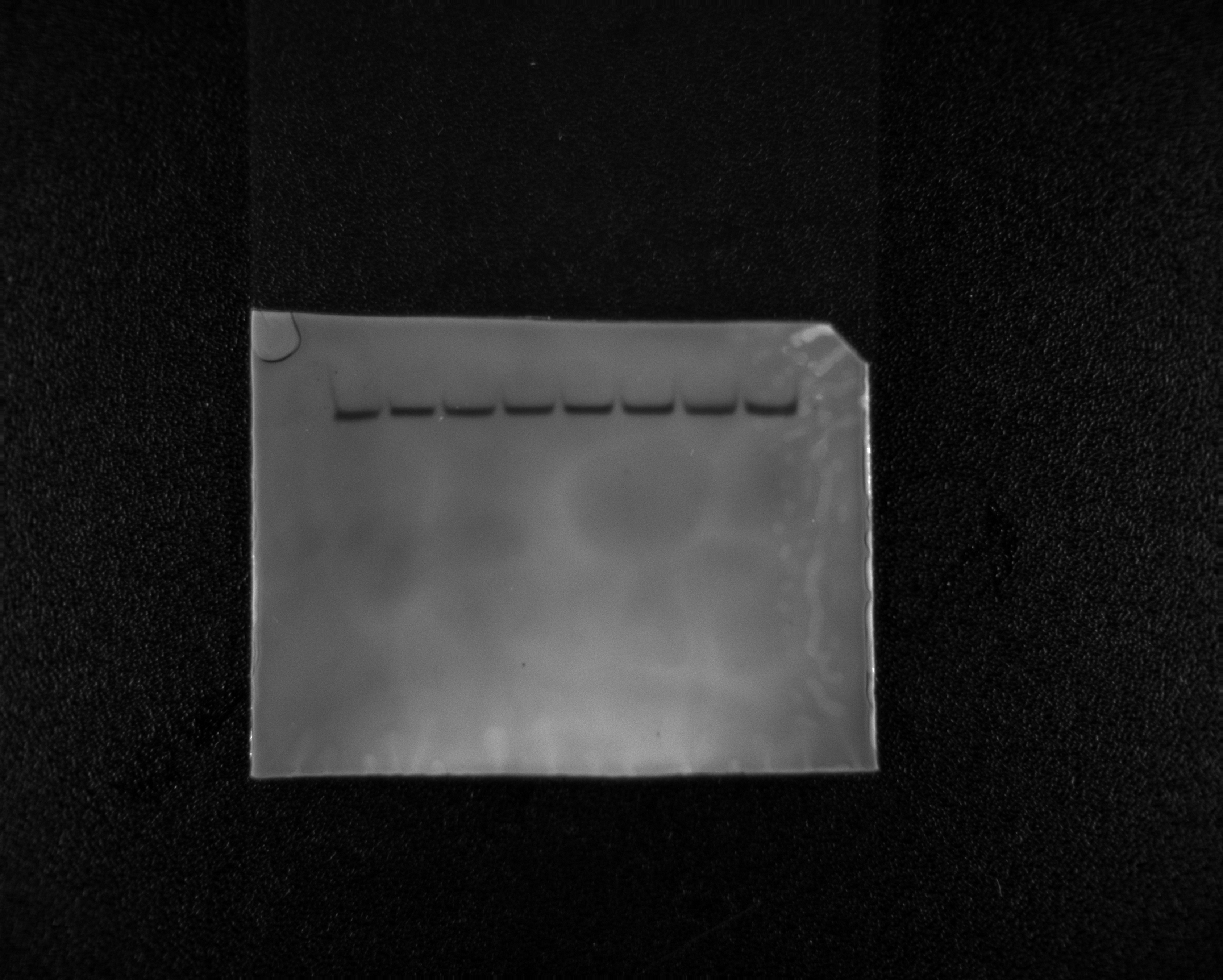

Supplement: Supplementary file 5 — Source data Fig. 3 [file 44319_2024_281_MOESM5_ESM.zip › Figure 3/3D/3D_UUAA.tif]

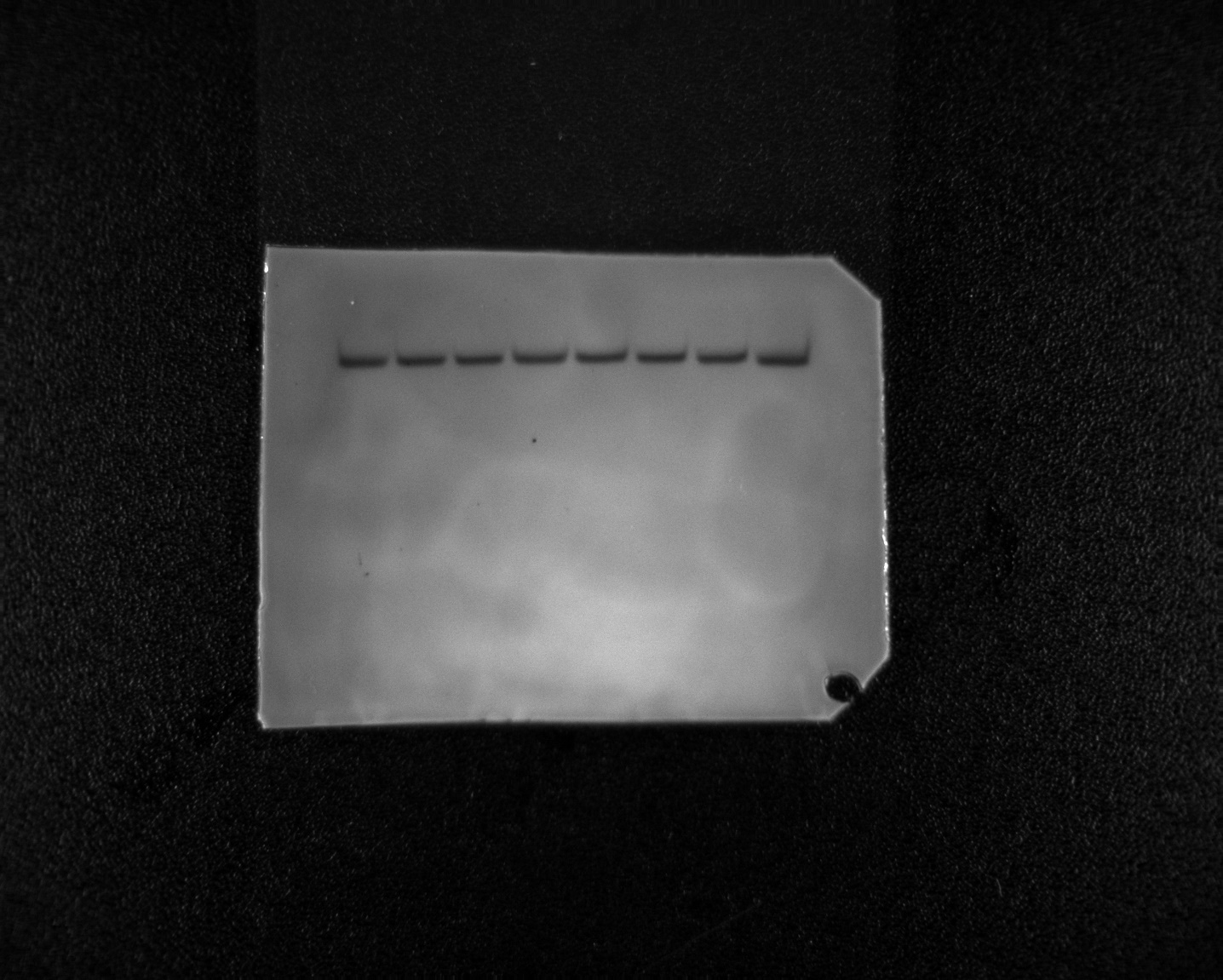


RNase T2 (ng/µl)

Loading control

0.05

0.1

0.25

0.5

1

2.5

5

Figure 3D

(dAdC)3UUGG(dAdC)4

Supplement: Supplementary file 5 — Source data Fig. 3 [file 44319_2024_281_MOESM5_ESM.zip › Figure 3/3D/3D_UUGG.docx]

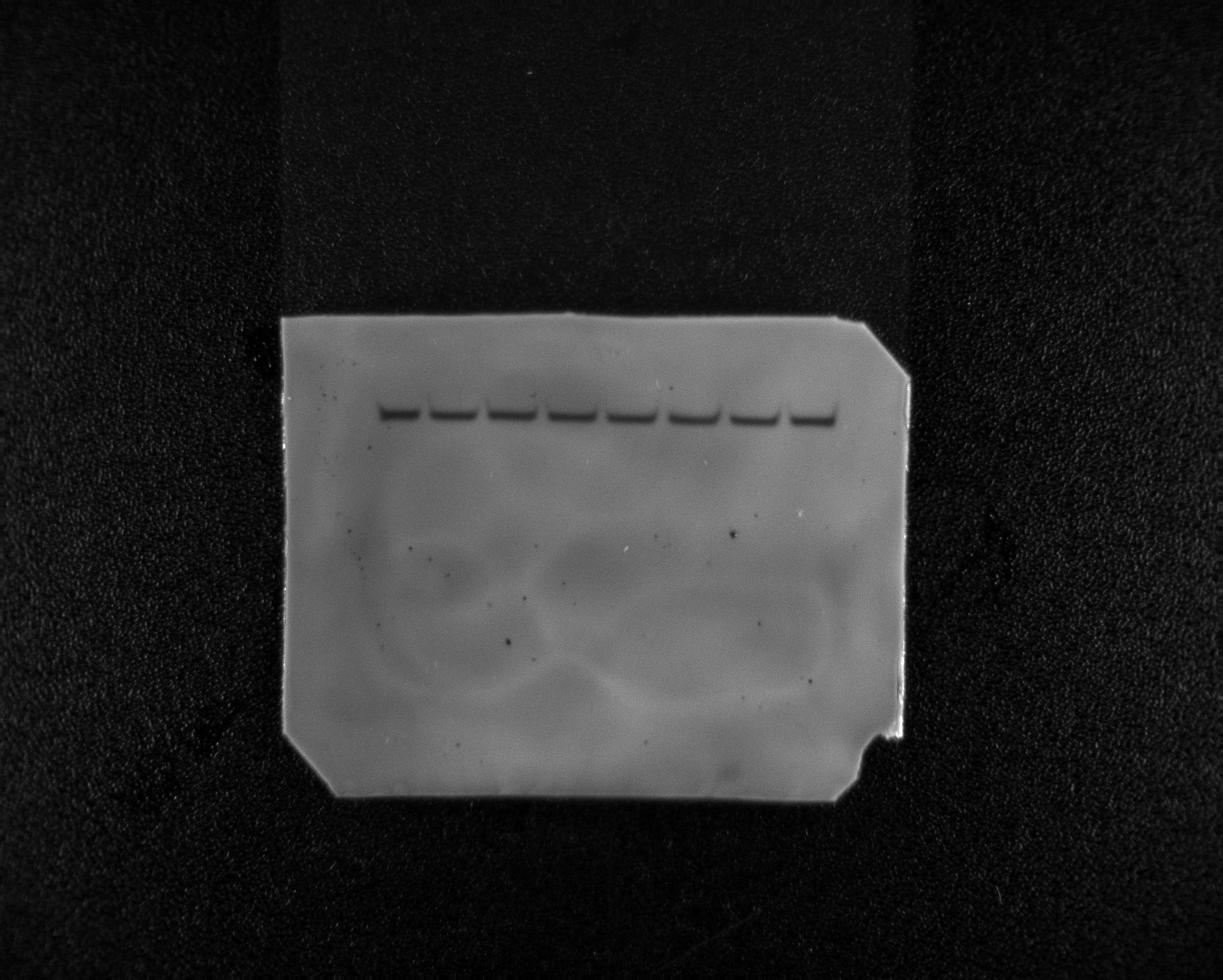

Supplement: Supplementary file 5 — Source data Fig. 3 [file 44319_2024_281_MOESM5_ESM.zip › Figure 3/3D/3D_UUUU.tif]

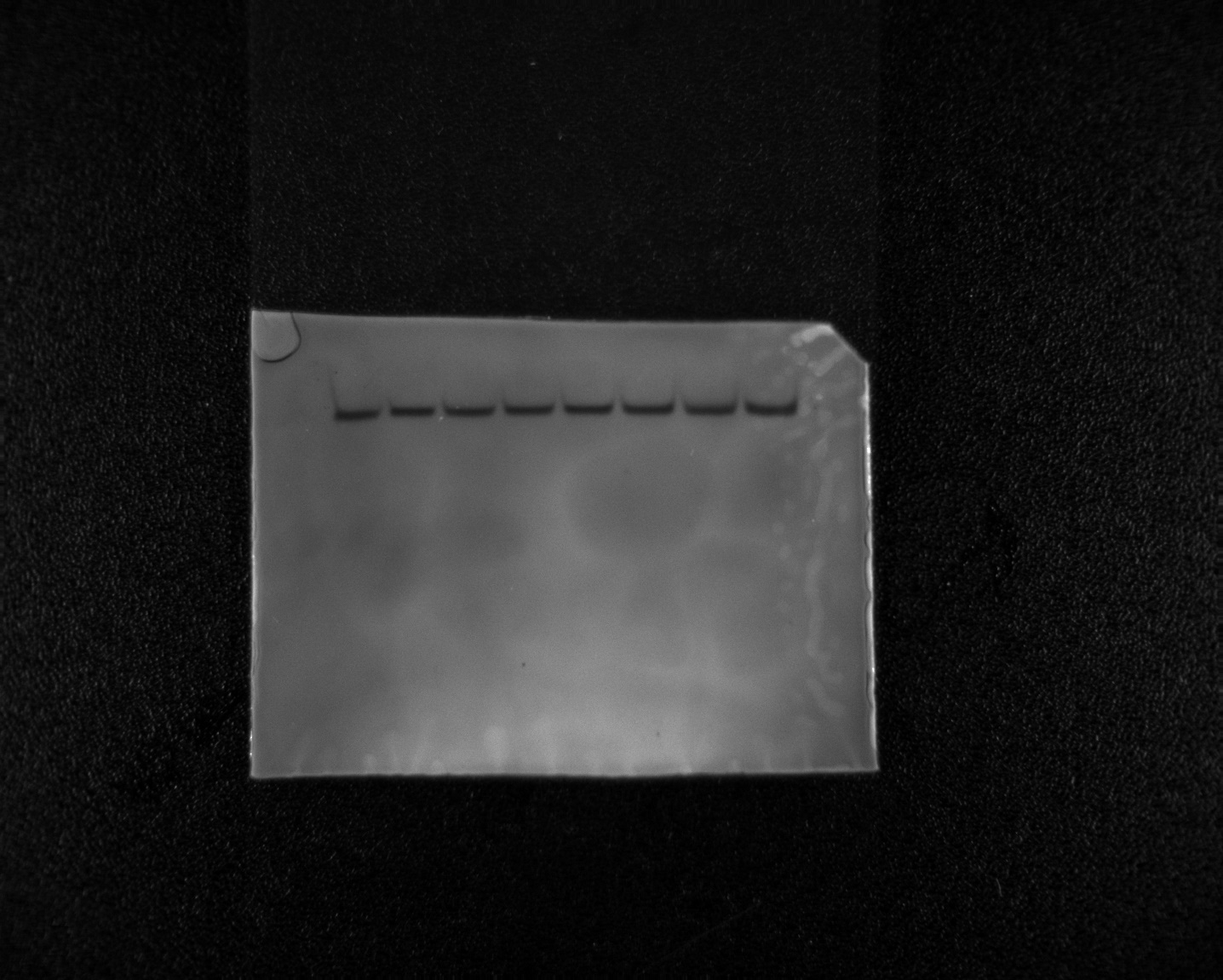


RNase T2 (ng/µl)

Loading control

0.05

0.25

0.1

5

2.5

1

0.5

(dAdC)3UUAA(dAdC)4

Figure 3D

Supplement: Supplementary file 5 — Source data Fig. 3 [file 44319_2024_281_MOESM5_ESM.zip › Figure 3/3D/3D_UUAA.docx]

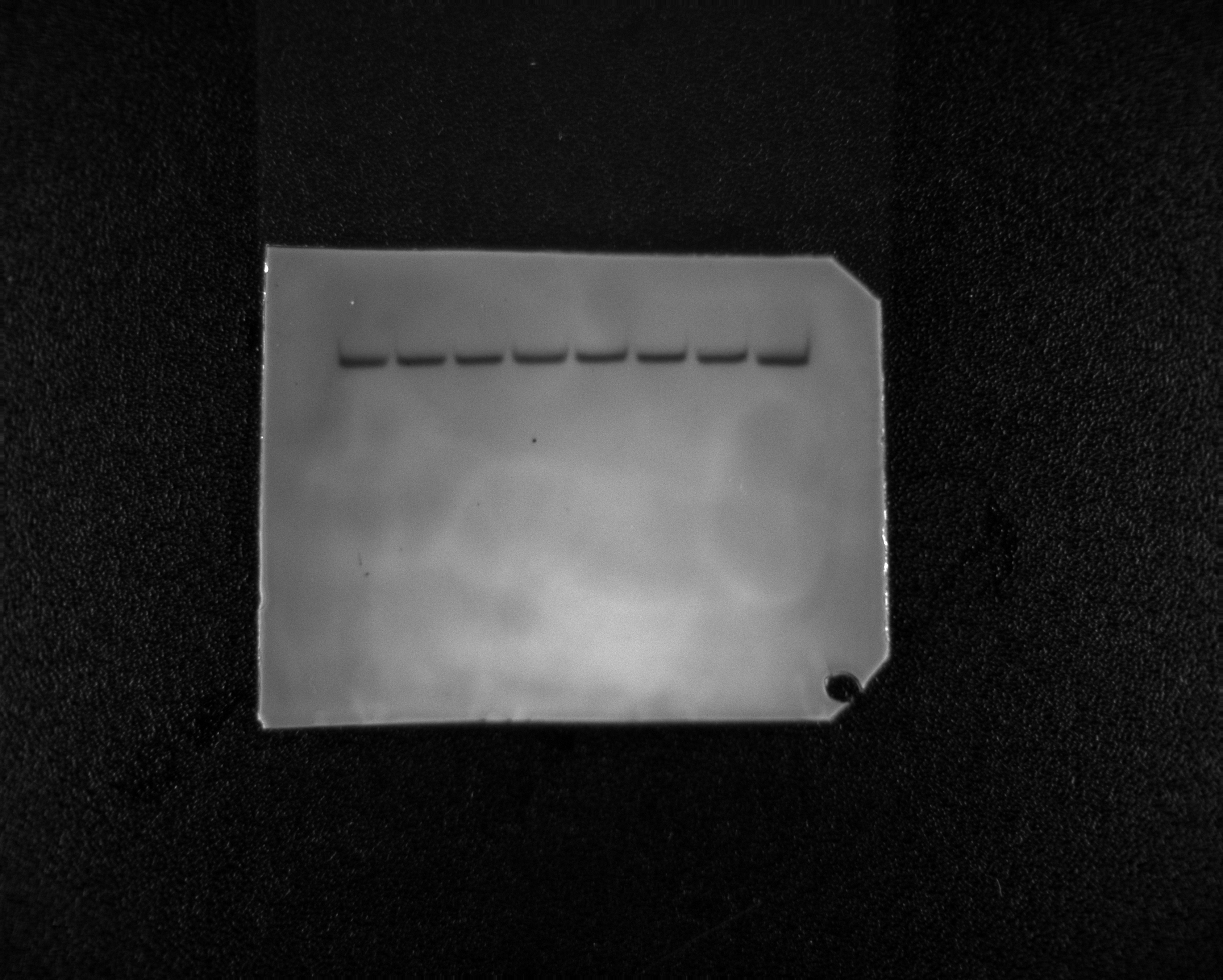

Supplement: Supplementary file 5 — Source data Fig. 3 [file 44319_2024_281_MOESM5_ESM.zip › Figure 3/3D/3D_UUGG.tif]

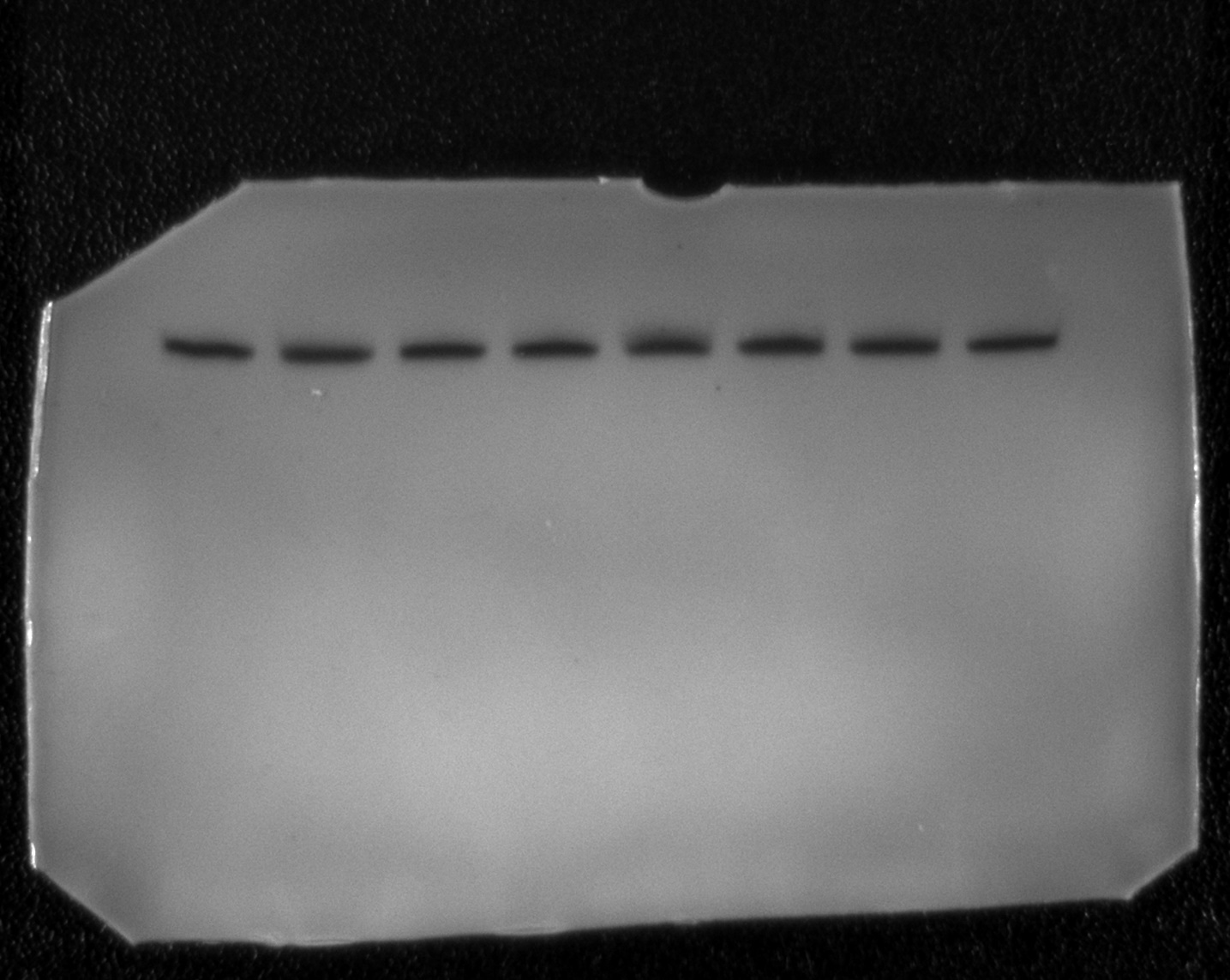


Loading control

0.05

0.1

0.25

0.5

1

2.5

5

(dAdC)3UUmAA(dAdC)4

Figure 4B

RNase 6 (ng/µl)

Supplement: Supplementary file 6 — Source data Fig. 4 [file 44319_2024_281_MOESM6_ESM.zip › Figure 4/4B/4B_UUmAA.docx]

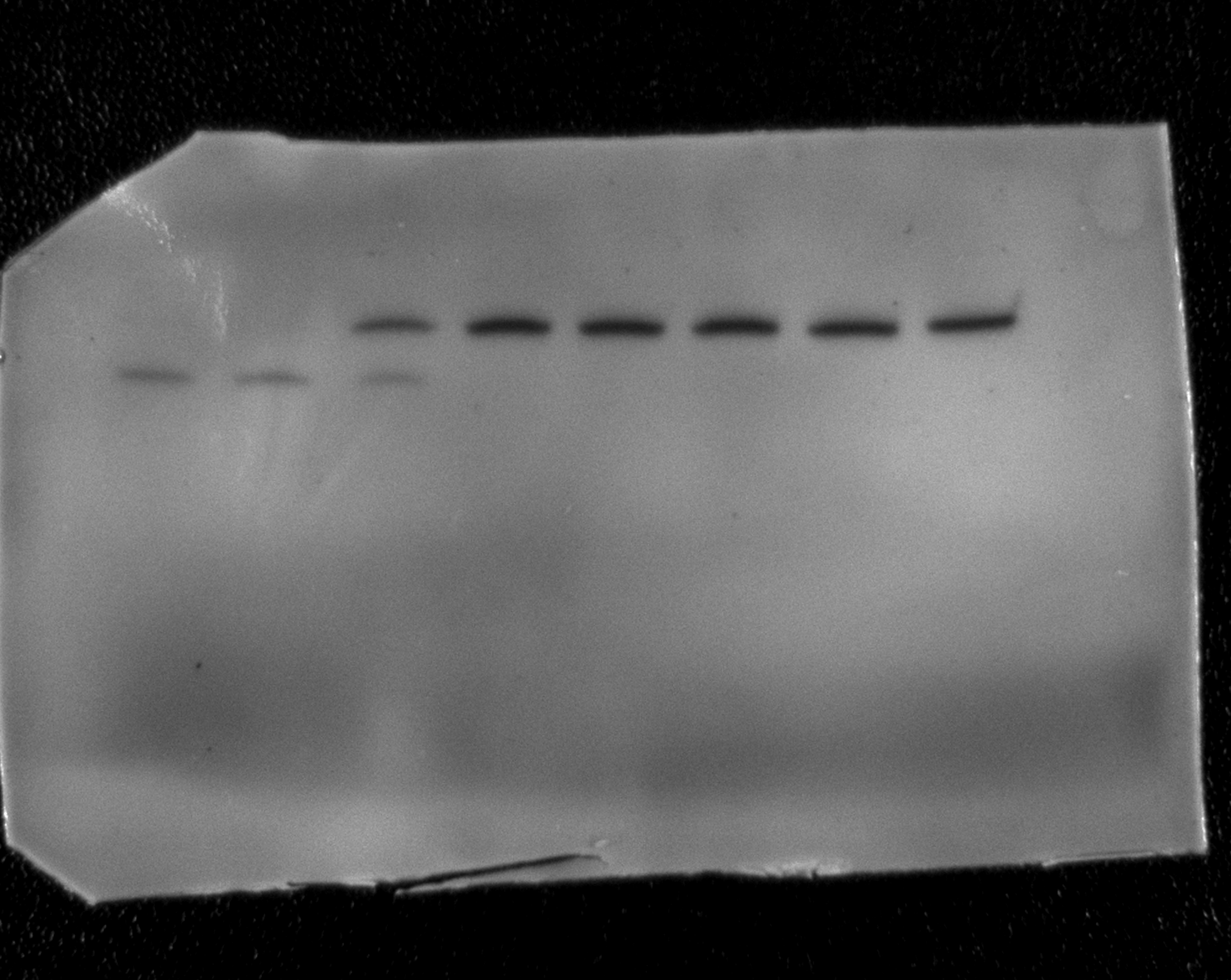

Supplement: Supplementary file 6 — Source data Fig. 4 [file 44319_2024_281_MOESM6_ESM.zip › Figure 4/4B/4B_UmUAA.tif]

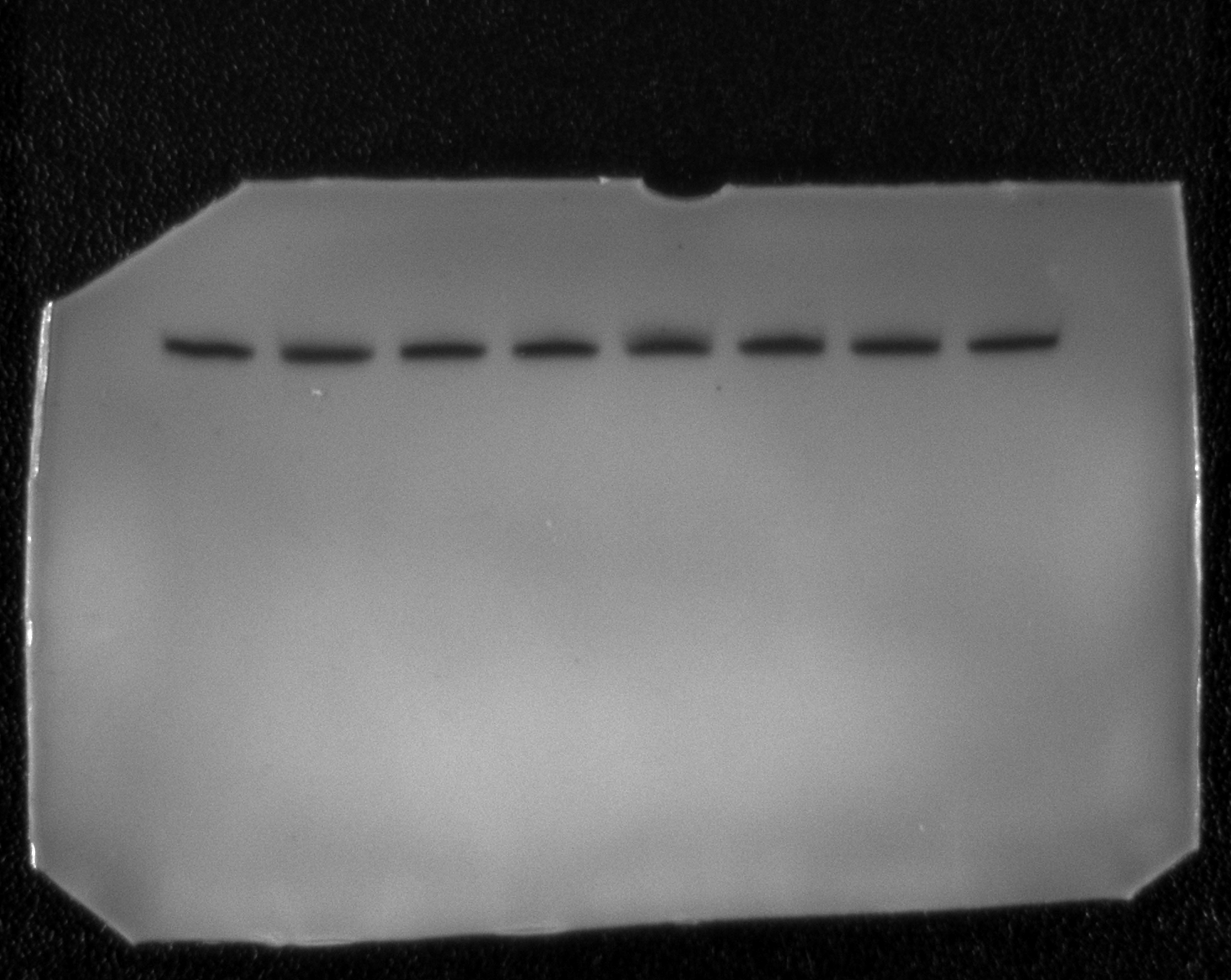

Supplement: Supplementary file 6 — Source data Fig. 4 [file 44319_2024_281_MOESM6_ESM.zip › Figure 4/4B/4B_UUmAA.tif]

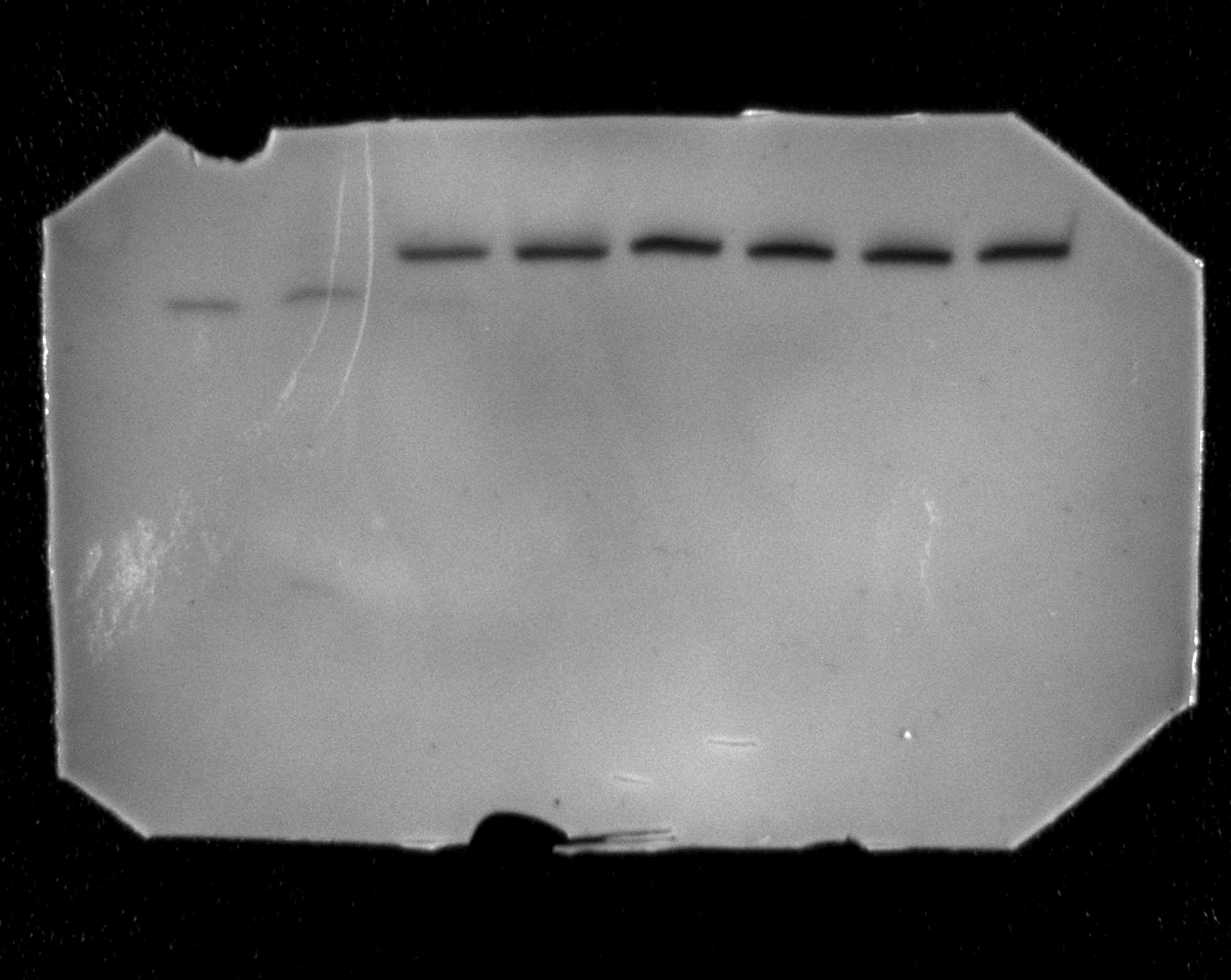

Supplement: Supplementary file 6 — Source data Fig. 4 [file 44319_2024_281_MOESM6_ESM.zip › Figure 4/4B/4B_UUAmA.tif]

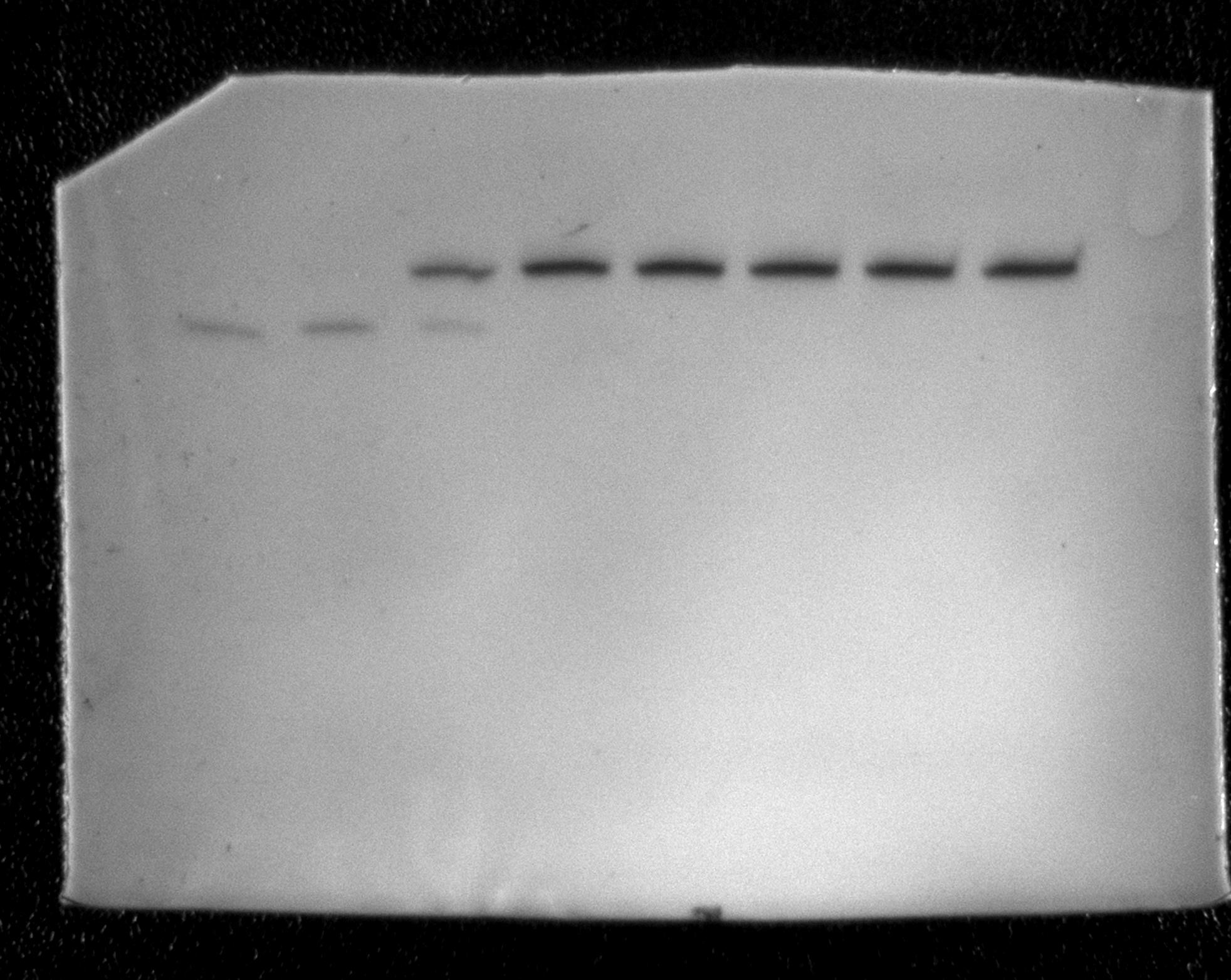

Supplement: Supplementary file 6 — Source data Fig. 4 [file 44319_2024_281_MOESM6_ESM.zip › Figure 4/4B/4B_UUAA.tiff]

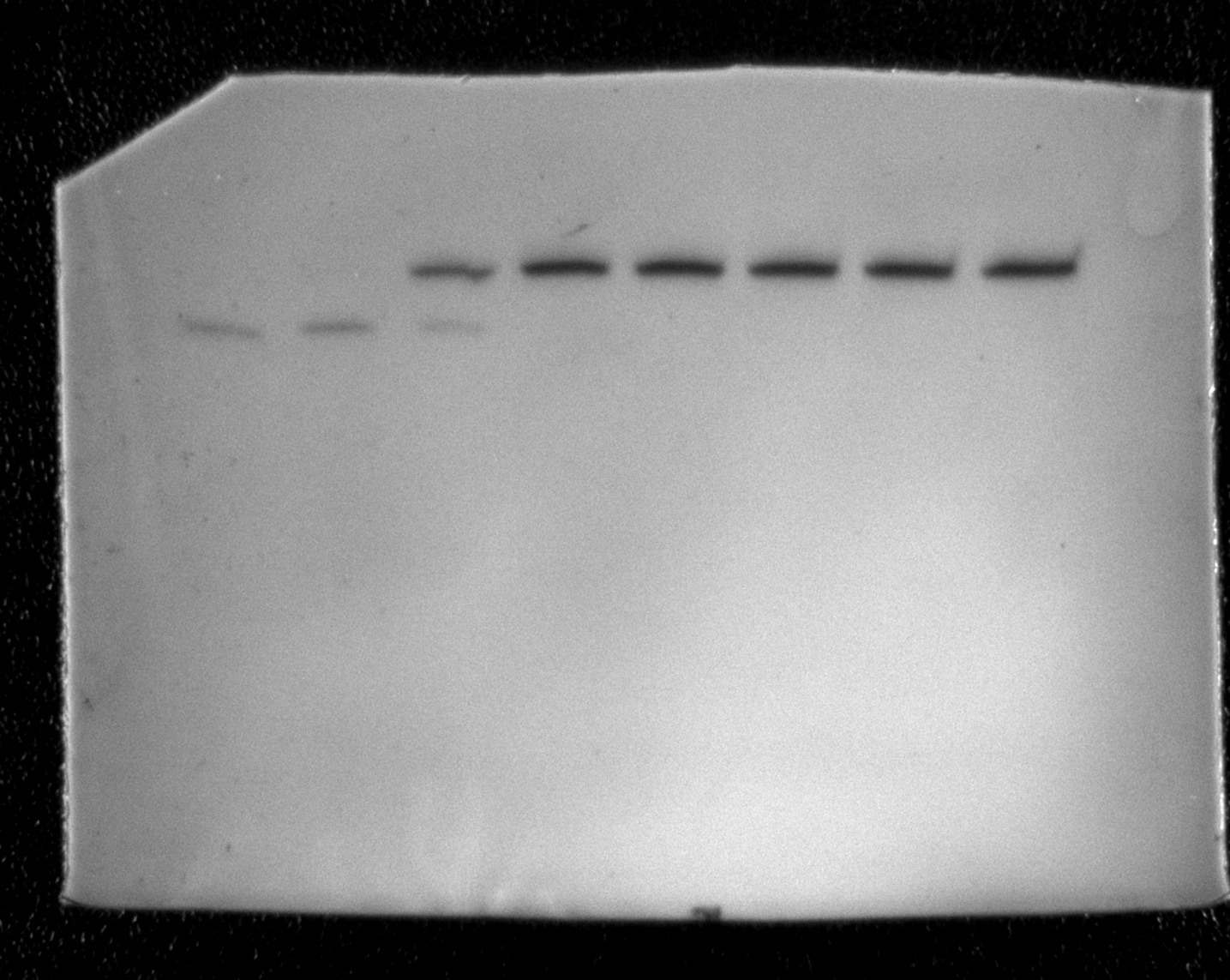


RNase 6 (ng/µl)

0.25

0.1

0.05

Loading control

0.5

1

2.5

5

(dAdC)3UUAA(dAdC)4

Figure 4B

Supplement: Supplementary file 6 — Source data Fig. 4 [file 44319_2024_281_MOESM6_ESM.zip › Figure 4/4B/4B_UUAA.docx]

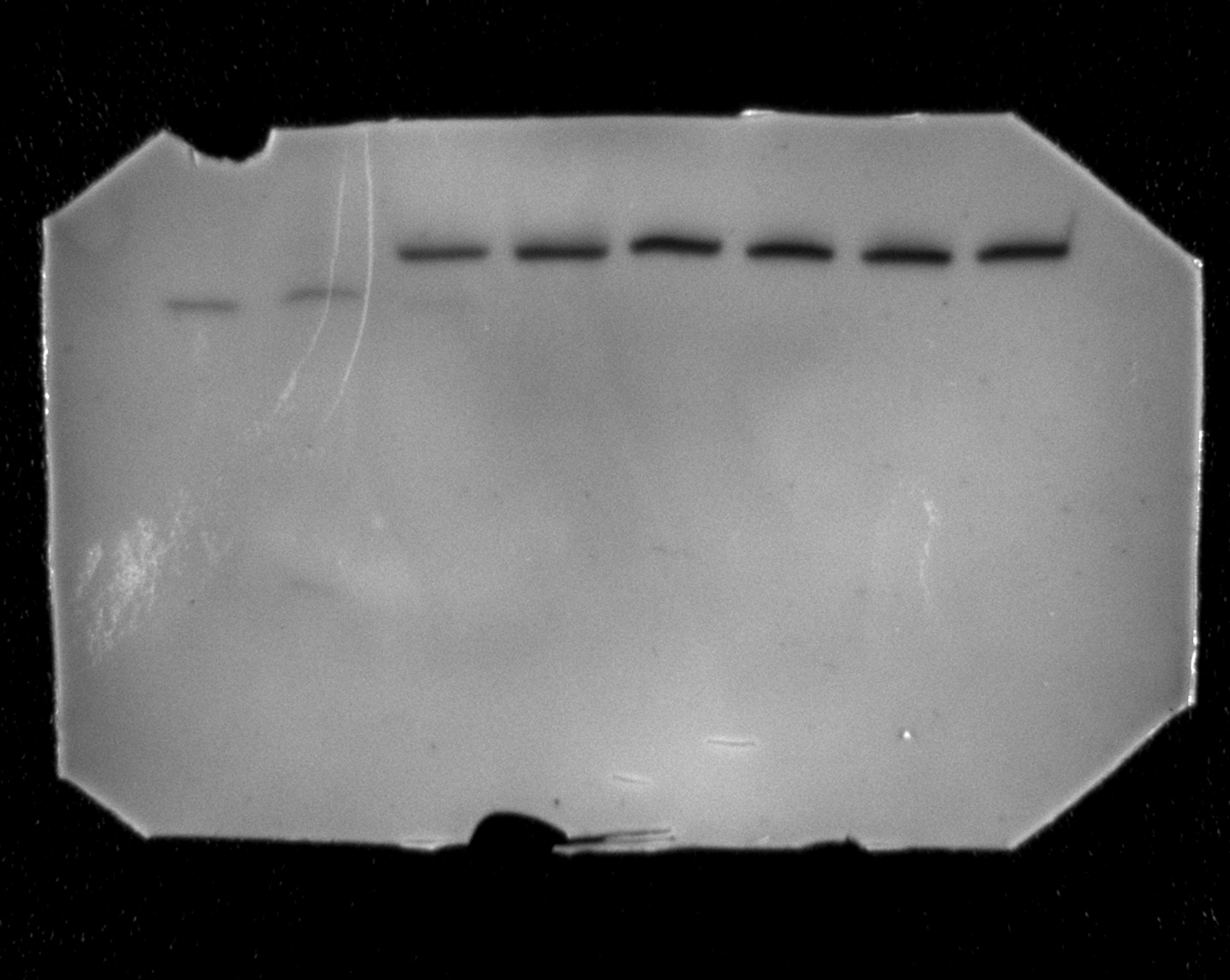


Loading control

0.05

0.1

0.25

0.5

1

2.5

(dAdC)3UUAmA(dAdC)4

Figure 4B

RNase 6 (ng/µl)

5

Supplement: Supplementary file 6 — Source data Fig. 4 [file 44319_2024_281_MOESM6_ESM.zip › Figure 4/4B/4B_UUAmA.docx]

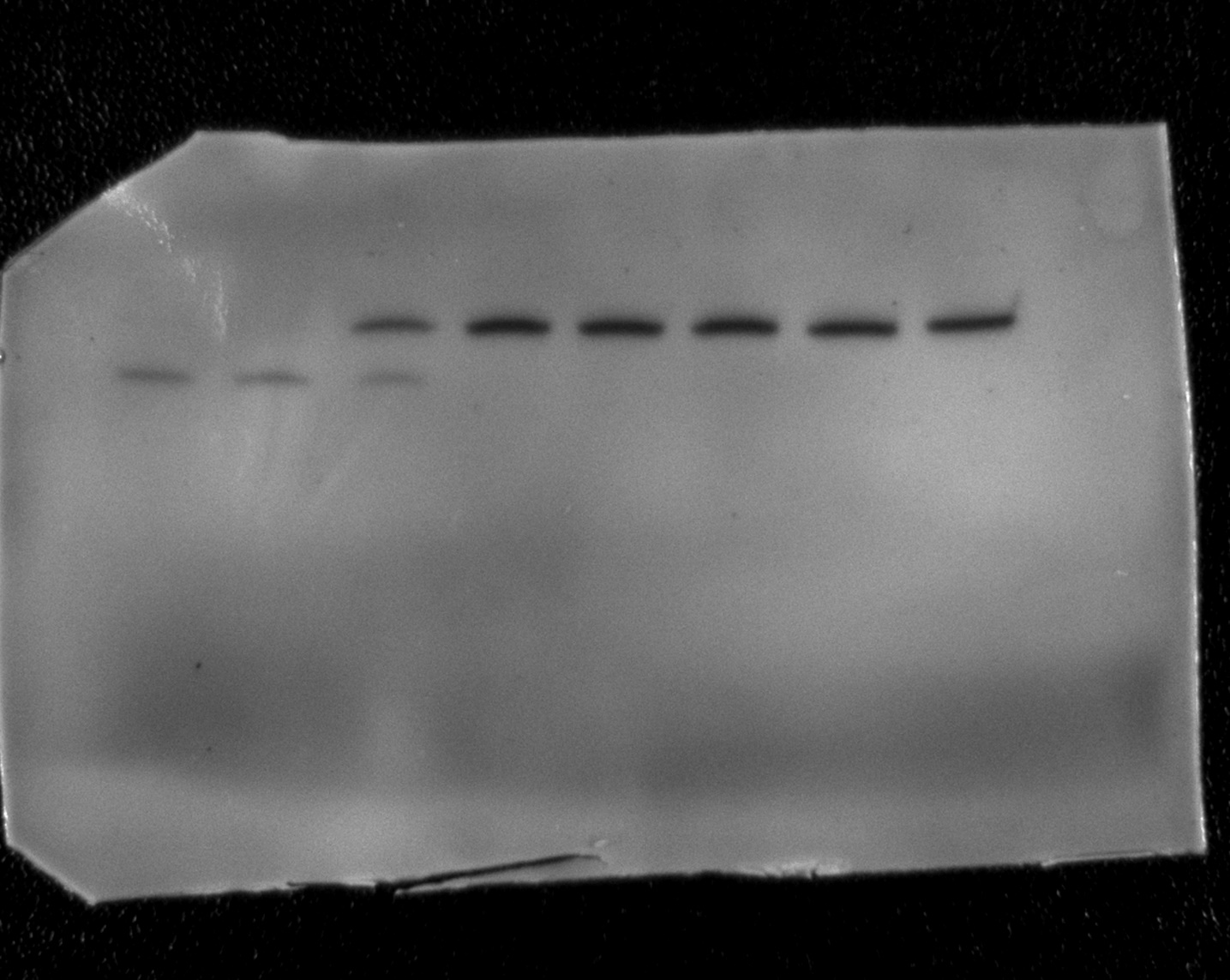


Loading control

0.05

0.1

0.25

0.5

2.5

1

5

(dAdC)3UmUAA(dAdC)4

Figure 4B

RNase 6 (ng/µl)

Supplement: Supplementary file 6 — Source data Fig. 4 [file 44319_2024_281_MOESM6_ESM.zip › Figure 4/4B/4B_UmUAA.docx]
