# Supplementary material for: Bacterial RNA sensing by TLR8 requires RNase 6 processing and is inhibited by RNA 2’O-methylation
Source: EMBO Rep. 2024 Oct 3;25(11):4674–92. doi: 10.1038/s44319-024-00281-9 (PMC11549399; doi:10.1038/s44319-024-00281-9)

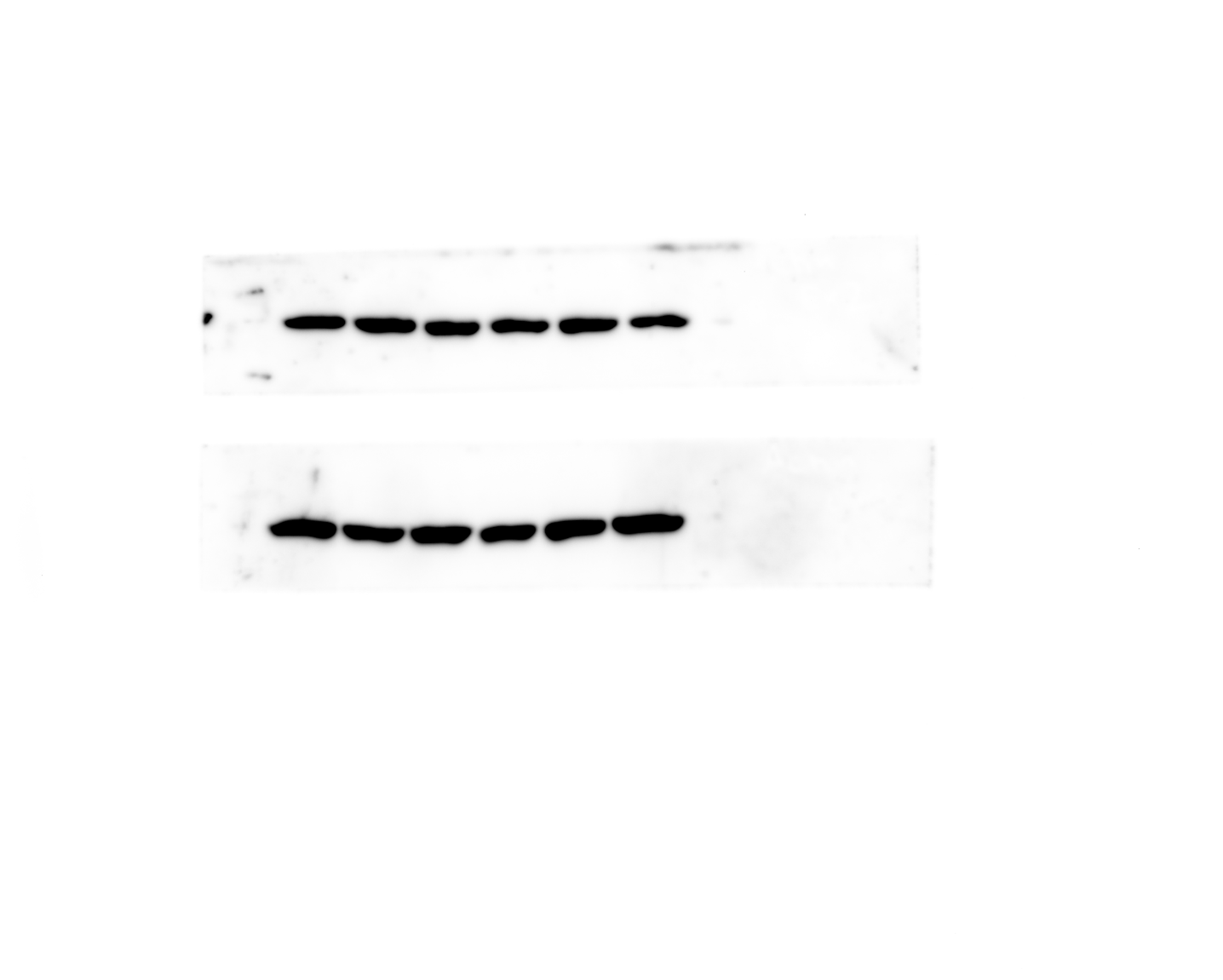


Marker

Marker

Experiment 2

Experiment 1

anti-β-Actin

35

35

50

50

sgRNASE6

sgRNASE6

sgRNASE6

sgRNASE6

sgRNASE6

sgRNASE6

Scramble

Scramble

Scramble

Scramble

Scramble

Scramble

kDa

Marker

Marker


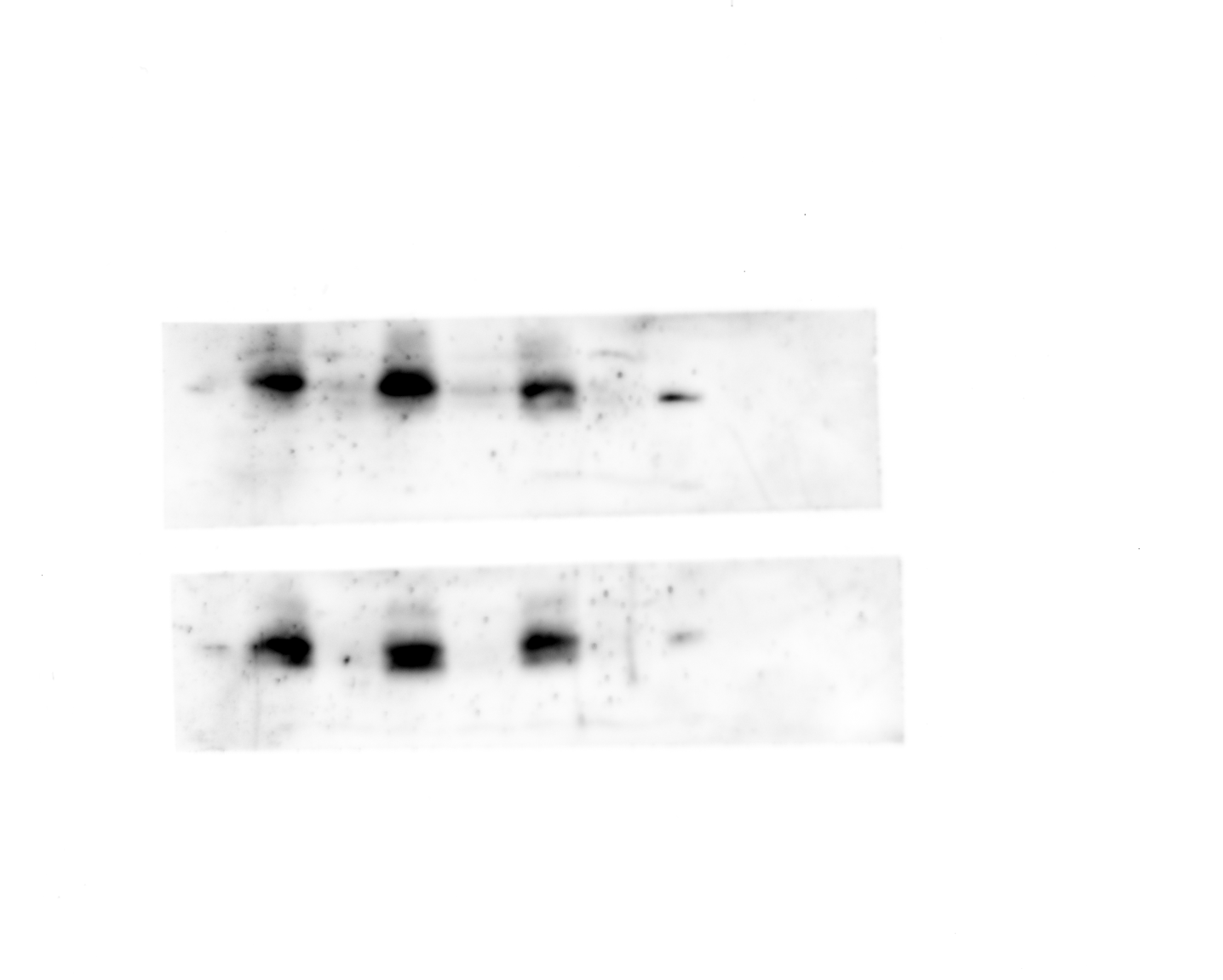


15

15

kDa

anti-RNase 6

Experiment 2

Experiment 1

Scramble

sgRNASE6

Scramble

sgRNASE6

Scramble

sgRNASE6

sgRNASE6

sgRNASE6

sgRNASE6

Scramble

Scramble

Scramble

Marker

Marker

Marker

Marker

Due to lack of responsiveness to TL8-506, a TLR8 specific agonist, one donor (Exp 1-2) shown in the blots was excluded from the set of TNF data in the Figures 2F/G/I and EV 2H. Values are shown below:

| Scramble | TL8-506 | TL8-506 |
| --- | --- | --- |
| Exp 1-1 | 979,9 | 138,75 |
| Exp 1-2 | 291,385 | 0 |
| Exp 1-3 | 4066,3 | 5628 |
| Exp 2-1 | 9236,5 | 7418 |
| Exp 2-2 | 15232 | 14573,5 |
| Exp 2-3 | 11789,5 | 12472 |

| sgRNASE6 | TL8-506 | TL8-506 |
| --- | --- | --- |
| Exp 1-1 | 873,7 | 334,65 |
| Exp 1-2 | 83,1 | 0 |
| Exp 1-3 | 5816,5 | 6950,5 |
| Exp 2-1 | 9077,5 | 8624,5 |
| Exp 2-2 | 16890 | 14766,5 |
| Exp 2-3 | 14254 | 12535 |


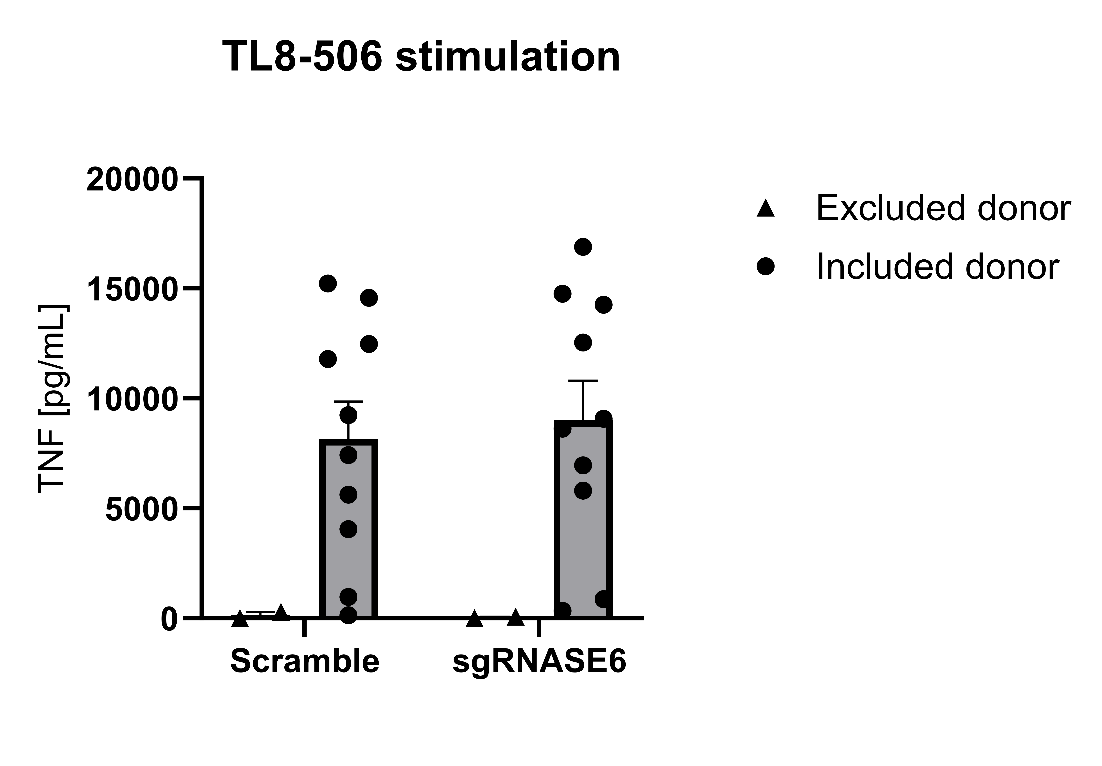

Supplement: Supplementary file 4 — Source data Fig. 2 [file 44319_2024_281_MOESM4_ESM.zip › Figure 2/2F/2F.docx]
