# Supplementary material for: Bacterial RNA sensing by TLR8 requires RNase 6 processing and is inhibited by RNA 2’O-methylation
Source: EMBO Rep. 2024 Oct 3;25(11):4674–92. doi: 10.1038/s44319-024-00281-9 (PMC11549399; doi:10.1038/s44319-024-00281-9)
Supplement: Supplementary file 4 — Source data Fig. 2 [file 44319_2024_281_MOESM4_ESM.zip › Figure 2/2B/Western blot_B-tubulin_RNase T2_RNase 6.docx]

*RNASE6*^-/-^

#3

#2

#1

*TLR8*^-/-^

Wild-Type

55

70

100

130

180

kDa

anti-β-Tubulin

Marker


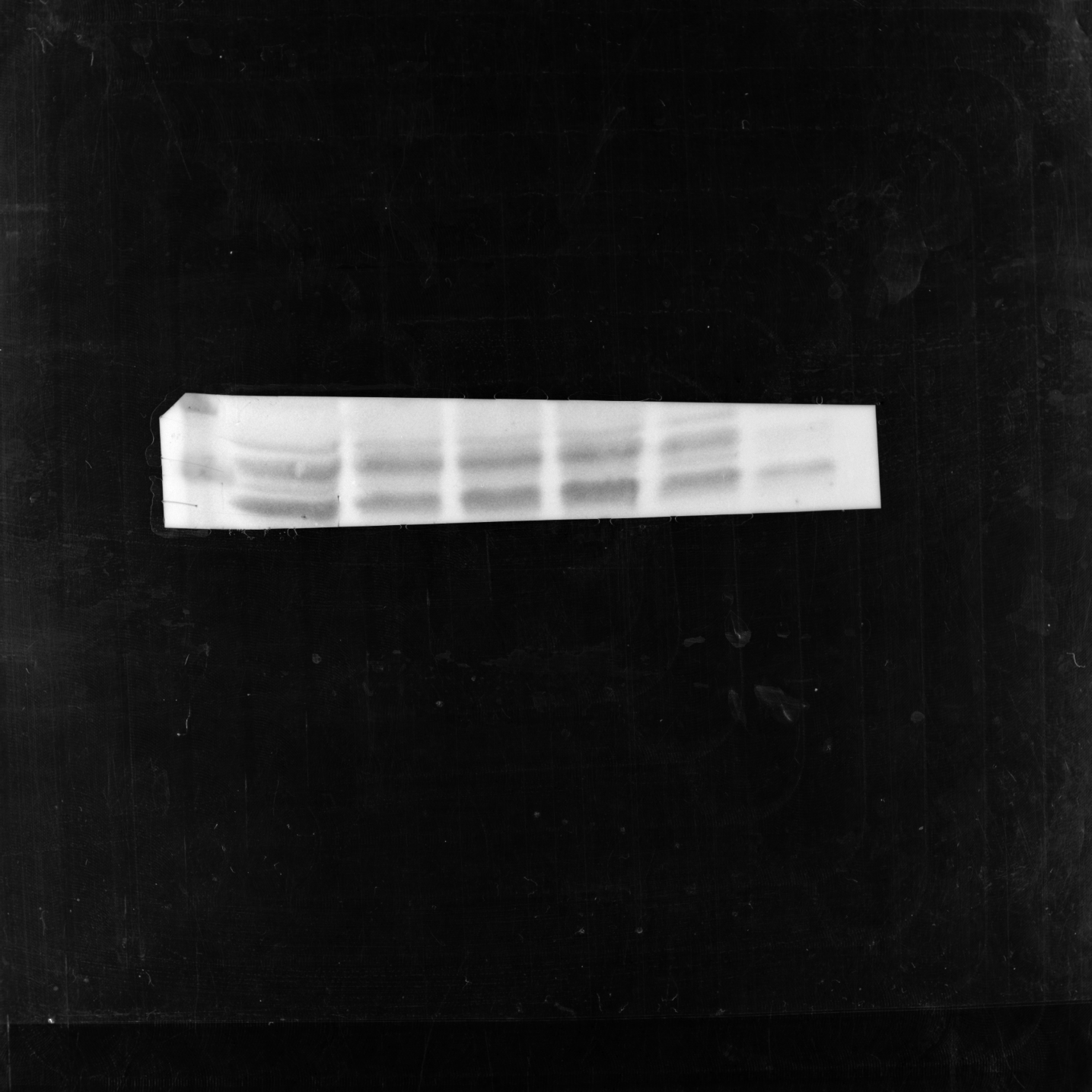


Wild-Type

#2

#3

*RNASE6*^-/-^

#1

*TLR8*^-/-^

anti-RNase T2 (only shown in the point to point rebuttal letter)

35

kDa

40

Marker


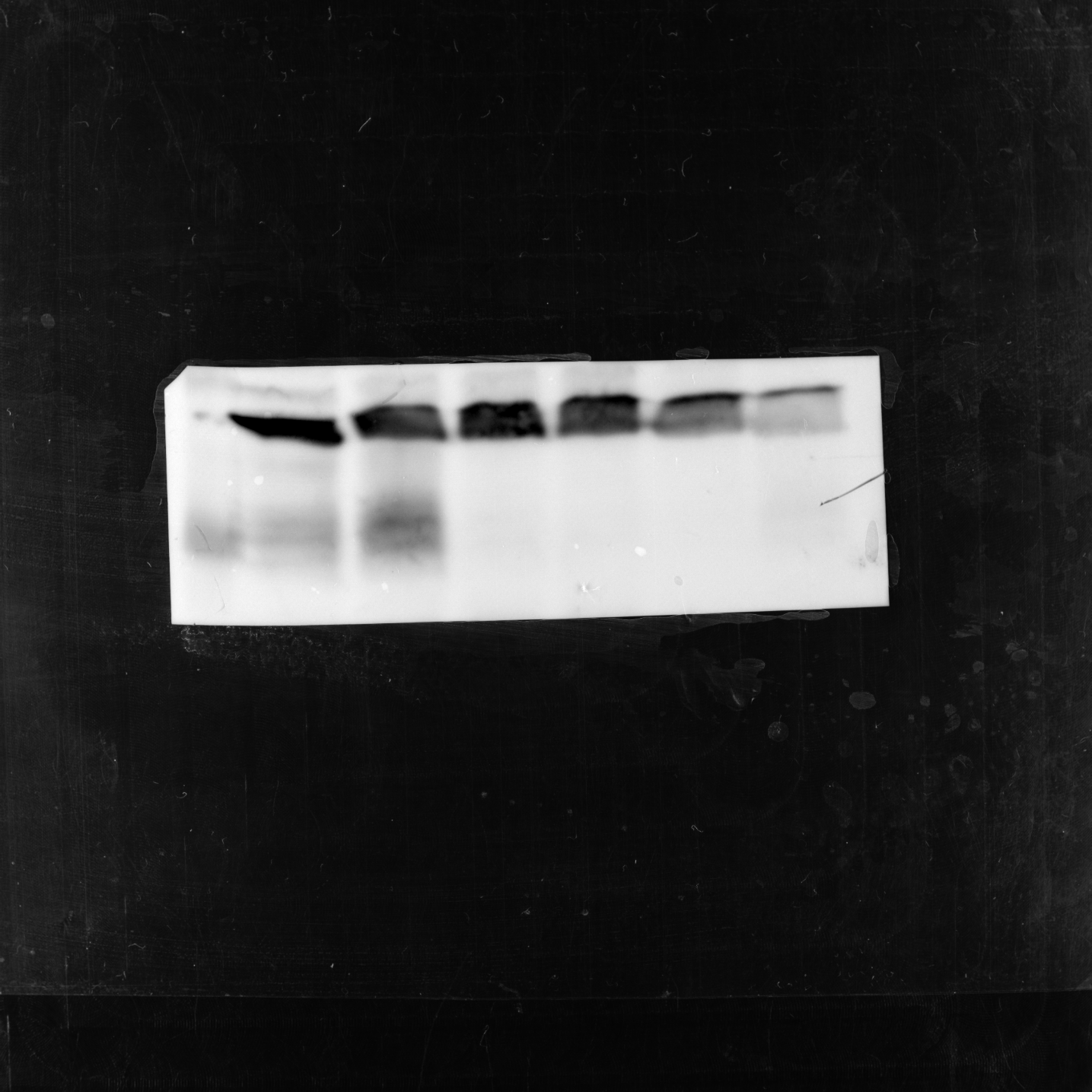


*RNASE6*^-/-^

#3

#2

#1

*TLR8*^-/-^

Wild-Type

Marker

15

25

kDa

anti-RNase 6
