# Supplementary material for: Bacterial RNA sensing by TLR8 requires RNase 6 processing and is inhibited by RNA 2’O-methylation
Source: EMBO Rep. 2024 Oct 3;25(11):4674–92. doi: 10.1038/s44319-024-00281-9 (PMC11549399; doi:10.1038/s44319-024-00281-9)
Supplement: Supplementary file 8 — Expanded View Figures [file 44319_2024_281_MOESM8_ESM.pdf]

Expanded View Figures

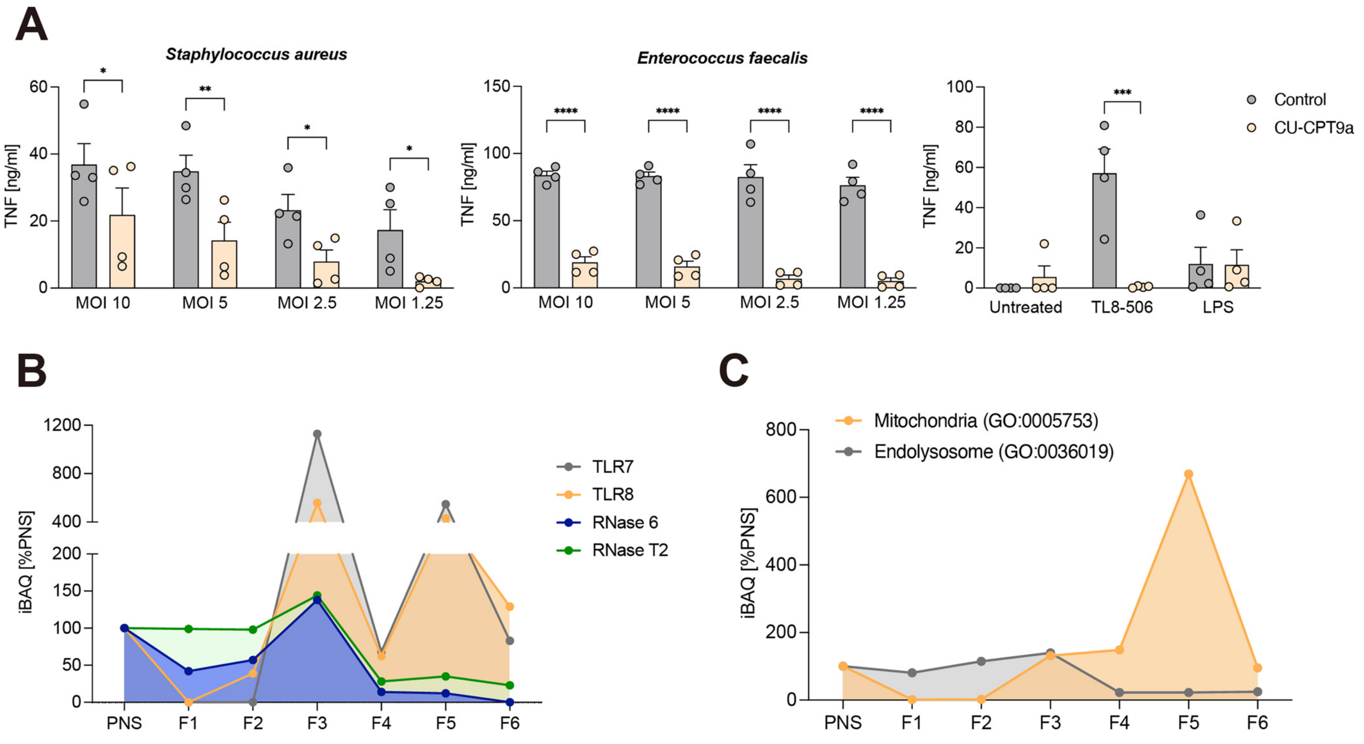

Figure EV1. ***RNASE6*** **is downregulated upon infection with TLR8-dependent bacteria.**

(**A**) Isolated monocytes were pre-treated with CU-CPT9a for 1 h, thereafter, cells were infected with different MOI. After 1 h, gentamycin was added to the wells. TNF-α was measured from the supernatant collected upon 20 h of incubation ($n = 2$, each with technical duplicates). Data were shown as mean ± SEM and analyzed by two-way ANOVA followed by Šídák's multiple comparisons test. Exact p values for individual sub-panels are reported here from left to right: *Staphylococcus aureus*, *$p = 0.0343$, **$p = 0.0042$, *$p = 0.0314$, and *$p = 0.0309$; *Enterococcus faecalis*, ****$p < 0.0001$; Controls, ***$p = 0.0001$. (**B**) Post-nuclear supernatant (PNS) of transdifferentiated BLaER1 cells was fractionated by ultracentrifugation on a sucrose gradient. From lowest to highest density, fractions 1–6 (F1–F6) were pooled from two gradients for in-gel digestion and analysis by HPLC-mass spectrometry to determine intensity-based absolute quantification (iBAQ) values. RNase 2 values were below the limit of detection. (**C**) The distribution of organelles over the density gradient as in (**B**) is shown exemplarily for mitochondria and endolysosomes. In detail, the iBAQ values of all identified proteins belonging to the gene ontology term GO:0036019 Endolysosome were used as average and normalized to the PNS. For mitochondria, the same was applied to all proteins associated with GO:0005753 mitochondrial proton-transporting ATP synthase complex. Source data are available online for this figure.

 © The Author(s)

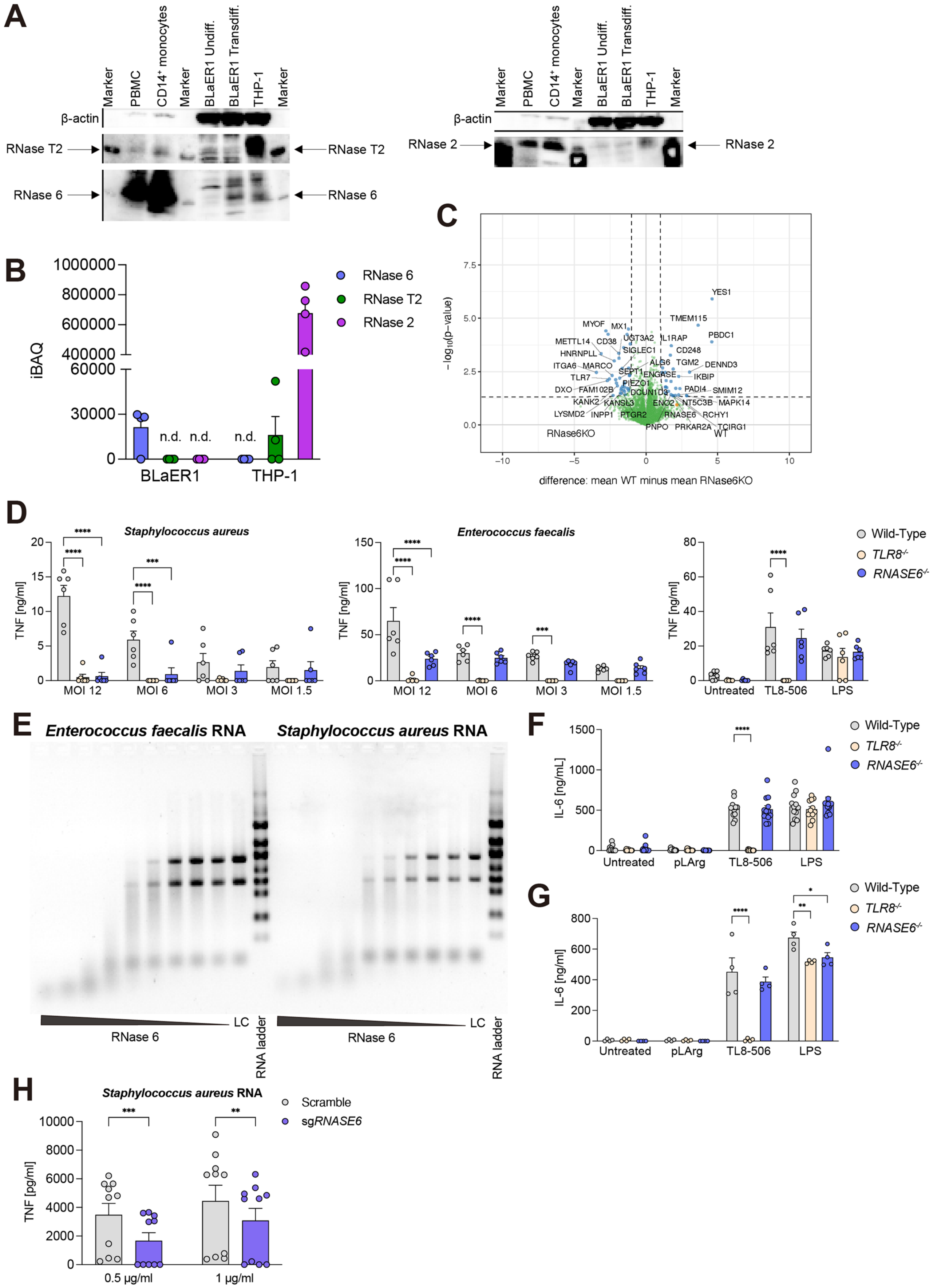
A
Marker
PBMC
CD14+ monocytes
BLaER1 Undiff.
BLaER1 Transdiff.
THP-1
β-actin
RNase T2
RNase 6
RNase 2
B
iBAQ
RNase 6
RNase T2
RNase 2
n.d.
BLaER1
THP-1
C
−log10(p-value)
difference: mean WT minus mean RNase6KO
RNase6KO
WT
YES1
TMEM115
PBDC1
D
Staphylococcus aureus
Enterococcus faecalis
TNF [ng/ml]
MOI 12
MOI 6
MOI 3
MOI 1.5
Untreated
TL8-506
LPS
Wild-Type
TLR8-/-
RNASE6-/-
E
Enterococcus faecalis RNA
Staphylococcus aureus RNA
RNase 6
LC
RNA ladder
F
IL-6 [ng/mL]
Untreated
pLArg
TL8-506
LPS
G
IL-6 [ng/ml]
H
Staphylococcus aureus RNA
Scramble
sgRNASE6
TNF [pg/ml]
0.5 μg/ml
1 μg/ml

Figure EV2. RNase 6 is required to generate TLR8 active RNA fragments from bacterial RNA.

(**A**) Immunoblot for RNase expression of the indicated cell types run with 25 µg of cell lysate. (**B**, **C**) Cellular proteomic analysis by HPLC-mass spectrometry to determine intensity-based absolute quantification (iBAQ) values comparing (**B**) BLaER1 and THP-1 cells or (**C**) Wild-type BLaER1 and *RNASE6*$^{-/-}$ cells from technical quadruplicates. The volcano plot was generated by R with the Welch *t*-test. The cutoff on the x-axis is for a twofold change and on the y-axis for a *p* value of 0.05. Measurements below the limit of detection are represented as not detected (n.d). (**D**) TNF-α detection by ELISA upon live whole bacterial infection with *S. aureus or E. faecalis* at several MOIs ($n = 3$, each with technical duplicates). Data were shown as mean ± SEM and analyzed with two-way ANOVA followed by Dunnett's test. Exact *p* values for individual sub-panels are reported here from left to right: *Staphylococcus aureus*, ****$p < 0.0001$ and ***$p = 0.0006$; *Enterococcus faecalis*, ****$p < 0.0001$ and ***$p = 0.0002$; Controls, ***$p = 0.0001$. (**E**) 1 µg of isolated bRNA was incubated with different concentrations of recombinant human RNase 6 (10 to 0.025 ng/µl) for 20 min and visualized by agarose gel. An untreated sample was loaded as a control (LC, loading control). Representative image out of two gels. (**F**) Controls of experiments are shown in panel 2D in main Fig. 2 ($n = 6$, each with technical duplicates). Data were shown as mean ± SEM and analyzed with two-way ANOVA followed by Dunnett's test. The exact *p* value is ****$p < 0.0001$. (**G**) Controls of predigested bRNA stimulation experiments are shown in panel 2E in main Fig. 2 ($n = 2$, each with technical duplicates). Data were shown as mean ± SEM and analyzed with two-way ANOVA followed by Dunnett's test. Exact *p* value are *$p = 0.0117$, **$p = 0.0022$, and ****$p < 0.0001$. (**H**) TNF-α detection upon *S. aureus* RNA stimulation in CD14+ monocytes edited by CRISPR-Cas9 ($n = 2$ in a total of five donors, each with technical duplicates). Data were shown as mean ± SEM and was analyzed with two-way ANOVA followed by Šídák's multiple comparisons test. The exact *p* values are **$p = 0.0064$ and ***$p = 0.0005$. pLArg poly-L-arginine, LPS lipopolysaccharide. Source data are available online for this figure.

 © The Author(s)

A

Label	Sequence
(dAdC)$_3$**GGAA**(dAdC)$_4$	dAdCdAdCdAdC**rGrGrArA**dAdCdAdCdAdCdAdC
(dAdC)$_3$**GGGG**(dAdC)$_4$	dAdCdAdCdAdC**rGrGrGrG**dAdCdAdCdAdCdAdC
(dAdC)$_3$**GGCC**(dAdC)$_4$	dAdCdAdCdAdC**rGrGrCrC**dAdCdAdCdAdCdAdC
(dAdC)$_3$**GGUU**(dAdC)$_4$	dAdCdAdCdAdC**rGrGrUrU**dAdCdAdCdAdCdAdC

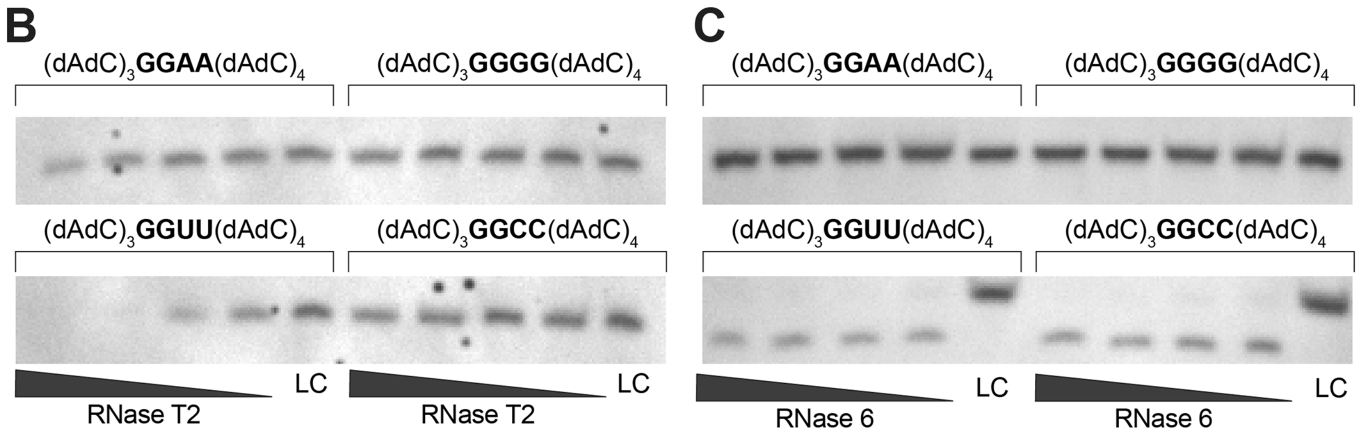

Figure EV3. RNase 6 cleavage releases uridine-terminated RNA fragments.

(**A**) Table of chimeric ORNs (protected deoxynucleotide sequences with central RNA) used for ex cellulo digestion by RNase 6 and RNase T2. (**B**) Urea gel of (dAdC)$_3$GGNN(dAdC)$_4$ digested by recombinant human RNase T2 or (**C**) RNase 6 (20 to 2.5 ng/µl). An untreated sample was loaded as a control (LC loading control). (**B**, **C**) Representative image out of two gels. N nucleotide, d deoxynucleotide, r ribonucleotide, A adenosine, G guanosine, U uridine, C cytosine). Source data are available online for this figure.

© The Author(s)
