# Supplementary material for: Bacterial RNA sensing by TLR8 requires RNase 6 processing and is inhibited by RNA 2’O-methylation
Source: EMBO Rep. 2024 Oct 3;25(11):4674–92. doi: 10.1038/s44319-024-00281-9 (PMC11549399; doi:10.1038/s44319-024-00281-9)
Supplement: Supplementary file 7 — EV Figures Source Data [file 44319_2024_281_MOESM7_ESM.zip › EMBOR-2024-59209V3-Figure_EV3_Source_Data-sd.zip!/Figure EV 3/EV 3B/EV 3B GGUU GGCC.docx]

(dAdC)_3_GGCC(dAdC)_4_

(dAdC)_3_GGUU(dAdC)_4_

RNase T2 (ng/µl)

RNase T2 (ng/µl)

Loading control

20

10

5

2,5

Loading control

2,5

5

10

20

Figure EV 3B
