# Supplementary material for: Bacterial RNA sensing by TLR8 requires RNase 6 processing and is inhibited by RNA 2’O-methylation
Source: EMBO Rep. 2024 Oct 3;25(11):4674–92. doi: 10.1038/s44319-024-00281-9 (PMC11549399; doi:10.1038/s44319-024-00281-9)
Supplement: Supplementary file 7 — EV Figures Source Data [file 44319_2024_281_MOESM7_ESM.zip › EMBOR-2024-59209V3-Figure_EV2_Source_Data-sd.zip!/Figure EV 2/EV 2E/EV 2E.docx]

200

500

10000

15000

20000

30000

40000

60000

Bases

RNase 6 (ng/µl)

RNase 6 (ng/µl)

0.025

10

10

0.025

5

2.5

1.25

0.5

0.25

0.1

0.05

5

2.5

1.25

0.5

0.25

0.1

0.05

Loading control

Loading control

Ladder

Ladder

*Staphylococcus aureus* RNA

*Enterococcus faecalis* RNA

Figure EV 2E
