# Supplementary material for: Bacterial RNA sensing by TLR8 requires RNase 6 processing and is inhibited by RNA 2’O-methylation
Source: EMBO Rep. 2024 Oct 3;25(11):4674–92. doi: 10.1038/s44319-024-00281-9 (PMC11549399; doi:10.1038/s44319-024-00281-9)
Supplement: Supplementary file 7 — EV Figures Source Data [file 44319_2024_281_MOESM7_ESM.zip › EMBOR-2024-59209V3-Figure_EV2_Source_Data-sd.zip!/Figure EV 2/EV 2A/EV 2A.docx]

Right part of panel

Left part of panel

35

35

50

50

PBMC

CD14+ monocytes

Marker

BLaER1 transdifferentiated

THP-1

Marker

Marker

Anti-β-Actin

BLaER1 undifferentiated

Figure EV 2A

kDa

kDa

Marker

Marker

PBMC

Marker

THP-1

BLaER1 transdifferentiated

BLaER1 undifferentiated

CD14+ monocytes

Anti-RNase T2

PBMC

Left part of panel

THP-1

BLaER1 transdifferentiated

CD14+ monocytes

BLaER1 undifferentiated

Marker

Marker

Marker

25

kDa

Anti-RNase 6

10

15

kDa

Left part of panel

THP-1

BLaER1 transdifferentiated

BLaER1 undifferentiated

CD14+ monocytes

Marker

Marker

Marker

PBMC

Anti-RNase 2

BLaER1 undifferentiated

BLaER1 transdifferentiated

THP-1

Marker

Marker

Right part of panel

Marker

PBMC

CD14+ monocytes

10

15

25

kDa
